# Pediatric Emergency Medicine Didactics and Simulation (PEMDAS): Serotonin Syndrome

**DOI:** 10.15766/mep_2374-8265.10928

**Published:** 2020-07-28

**Authors:** Corinne Shubin, Shweta Iyer, Jean Pearce, Benjamin Lang, Isabel Gross, Daisy Ciener, Suzan Mazor, Ashley Keilman, Anita Thomas

**Affiliations:** 1 Assistant Professor, Department of Pediatrics, Division of Emergency Medicine, University of Washington School of Medicine and Seattle Children's Hospital; 2 Assistant Professor, Pediatrics and Emergency Medicine, Weill Cornell Medical College; 3 Assistant Professor, Department of Pediatrics, Division of Emergency Medicine, Medical College of Wisconsin; 4 Pediatrics Resident, Department of Pediatrics, University of Washington School of Medicine and Seattle Children's Hospital; 5 Clinical Instructor, Department of Pediatrics, Division of Emergency Medicine, Yale University School of Medicine and Yale New Haven Children's Hospital; 6 Assistant Professor, Clinical Pediatrics, Division of Pediatric Emergency Medicine, Vanderbilt University Medical Center; 7 Director of Pediatric Toxicology, University of Washington School of Medicine and Seattle Children's Hospital; Associate Professor, Department of Pediatrics, Division of Emergency Medicine, University of Washington School of Medicine and Seattle Children's Hospital

**Keywords:** Serotonin Syndrome, Simulation, Rhabdomyolysis, Seizure, Altered Mental Status, Pediatric Emergency Medicine, Emergency Medicine, Clonus, Hyperthermia

## Abstract

**Introduction:**

Serotonin syndrome is caused by an accumulation of serotonin in the body from drug interactions or overdose of serotonergic medications, including commonly used antidepressants. Symptoms can be life-threatening and encompass both neurologic and cardiovascular toxicity, including agitation, seizure, tachycardia, rhabdomyolysis, and hyperthermia.

**Methods:**

This simulation case was developed for pediatric emergency medicine fellows and emergency medicine residents in the pediatric emergency department and can be altered to accommodate other learners. The case involved a 16-year-old male, represented by a low- or high-fidelity manikin, who presented with altered mental status/agitation after an overdose of antidepressant medication. The team of learners was required to perform a primary and a secondary assessment; manage airway, breathing, and circulation; and recognize and initiate treatment for serotonin syndrome. The patient had a seizure resulting in airway compromise requiring advanced airway support, as well as developed rhabdomyolysis requiring aggressive fluid hydration. We created a debriefing guide and a participant evaluation form.

**Results:**

Fifty-seven participants across five institutions completed this simulation, which included residents, fellows, faculty, and students. The scenario was rated by participants using a 5-point Likert scale and was generally well received. Participants rated the simulation case as effective in learning how to both recognize (*M* = 4.9) and manage (*M* = 4.8) serotonin syndrome.

**Discussion:**

This pediatric emergency simulation scenario can be tailored for a range of learner backgrounds and simulation environments. We used the participant evaluation form to improve future iterations of the simulation.

## Educational Objectives

By the end of this activity, learners will be able to:

1. Demonstrate the ability to assess and emergently manage a pediatric patient with agitation, altered mental status, and disability, as well as to perform frequent reassessments.
2. Identify a possible toxicologic ingestion and formulate a differential, including serotonin syndrome.
3. Develop and execute a management plan for a patient with serotonin syndrome.
4. Identify and treat rhabdomyolysis.
5. Demonstrate effective team leadership, roles, and communication.

## Introduction

Serotonin syndrome is a potentially life-threatening medical emergency that is caused by excessive serotonergic activity. There are no laboratory tests to confirm the diagnosis of serotonin syndrome; thus, clinicians must be able to recognize the signs and symptoms and take an accurate medication history in order to correctly make this clinical diagnosis.^1,2^ Serotonin syndrome manifests as a combination of mental status changes, autonomic instability, and neuromuscular hyperactivity.^1,2^ Symptoms of excessive serotonergic activity range from mild, such as tremor and akathisia, to progressively more severe, such as clonus, altered mental status, hypertonicity and rigidity, hyperthermia, and seizure.^2^ Hunter serotonin toxicity criteria are used to identify and diagnose serotonin syndrome, which require one or more of the following classic findings in the presence of a serotonergic agent to make the diagnosis: spontaneous clonus alone, inducible or ocular clonus with either agitation or diaphoresis, tremor with hyperreflexia, or hypertonicity and hyperthermia with ocular or inducible clonus.^3^

Management of serotonin syndrome involves discontinuing the precipitating agent(s), providing supportive care, controlling autonomic instability, and controlling hyperthermia.^2^ The use of benzodiazepines is central to controlling agitation, tremor, hypertonicity, and hyperthermia.^1,2^ Benzodiazepines should be dosed to effect and given as frequently as needed for symptomatic management. Eliminating excessive muscle activity is critical to management of hyperthermia. Seizures may occur with severe serotonin syndrome and have been reported in overdoses of various selective serotonin reuptake inhibitors (SSRIs). Citalopram overdoses have been associated with more severe intoxications and higher rates of seizures than have other SSRIs; however, seizures have also been reported in overdoses with SSRIs that are considered more benign, such as fluoxetine.^4^ There is limited data regarding the efficacy of serotonergic antagonist therapy in serotonin syndrome.^2^ Clinicians may consider a dose of cyproheptadine, a 5-HT2A antagonist; however, this medication is only available in oral form and may not be appropriate to administer given the clinical situation with altered mental status. Clinicians desiring a parenteral serotonergic antagonist may try a dose of chlorpromazine; however, this is considered outdated and there are little data supporting its use.^2^ A creatinine kinase (CK) level should be checked in patients with severe serotonin syndrome on presentation as rhabdomyolysis, as this may indicate a potential complication of severe serotonin syndrome resulting from excessive muscle activity.^1,2^ Prevention of acute kidney injury focuses on aggressive fluid resuscitation. Electrolytes should be monitored closely, and electrolyte derangements may need correction, with particular attention paid to the potassium level and risk for arrhythmia.

This simulation-based curriculum allows learners to perform essential pediatric emergency medicine resuscitation skills, practice effective team leadership/communication, discuss a broad differential for managing a patient with altered mental status, and demonstrate treatment of serotonin syndrome. The simulation was developed by several content experts in pediatric emergency medicine from all five participating sites and included a pediatric toxicology content expert (Suzan Mazor). In utilizing Kirkpatrick's Model of learning,^5^ this curriculum sets the groundwork for reaction and learning. The evaluation form provided in the package and the in-person debriefing allow for real-time reactions by learners as well as allow facilitators to assess what was learned by asking pointed, knowledge-based questions on serotonin syndrome. The simulation was not designed for individual assessment, but rather for formative assessment of a team of learners.

Serotonin syndrome is an increasingly common and life-threatening condition seen in pediatric emergency medicine departments, and it is important for medical providers to recognize and manage serotonin syndrome expediently and in a team setting. Although team interaction is difficult to assess, this simulation provides a valid medium to assess team dynamics, communication, and knowledge, as well as may elucidate systems issues. Depending on learners' needs, this curriculum may be used with other simulation-based curricula from the Pediatric Emergency Medicine Simulation Curriculum series^6–22^ or the Pediatric Toxidrome Simulation Series,^23–27^ or the curriculum may be used independently. This curriculum was originally developed for pediatric emergency medicine fellows and general emergency medicine residents and would also be appropriate for family medicine and pediatrics residents as well as interdisciplinary simulations involving pediatric emergency physicians, fellows, residents, medical students, nurses, nurse practitioners, pharmacists, and respiratory therapists. Prior to our development of this simulation scenario, there were no simulation curricula in *MedEdPORTAL* covering a case of serotonin syndrome, and thus we present this scenario as a unique contribution to the literature.

## Methods

### Development

We designed this simulation case (Appendix A) to help learners recognize and manage various features of serotonin syndrome, or hyper-serotonergic state, including hyperthermia, hypertonia, seizures, and rhabdomyolysis. Additionally, this case allowed participants to further develop teamwork and communication skills. Through participation in this scenario, learners demonstrated how to perform an initial patient assessment, recognize altered mental status, apply monitors, gain appropriate intravenous access, and obtain pertinent history from the patient's parent. The learners recognized concern for toxic ingestion, specifically serotonin syndrome, and demonstrated appropriate management of agitation, clonus, and rigidity with administration of benzodiazepines, as well as appropriate management of hyperthermia with cooling methods and of seizure with securing the airway. Learners assigned roles, including group leaders, and demonstrated effective communication skills.

The equipment preparation list (Appendix B) was put together by content experts in pediatric emergency medicine at the five participating institutions and was edited based on learner feedback about the simulation. The critical action checklist (Appendix C) was also put together by pediatric emergency medicine content experts from all participating institutions, as well as a pediatric toxicologist (Suzan Mazor) from one participating institution. This checklist was vetted by all sites prior to running the first simulation. Appendices D and E are an electrocardiogram and a chest X-ray that were provided by the primary author (Corinne Shubin). The debriefing guide (Appendix F) and the teamwork and communication glossary (Appendix G) were modeled after standard simulation debriefing tools and commonly used health care terminology. Appendix H was collated by pediatric emergency medicine content experts and the toxicology content was checked by a pediatric toxicologist (Suzan Mazor). The primary and senior authors (Corinne Shubin and Anita Thomas) developed the evaluation form (Appendix I) based on the learning objectives, and the evaluation form was approved by all five participating sites prior to the first simulation iteration.

We implemented this resource for learners during pediatric emergency medicine teaching sessions. These sessions ranged from scheduled weekly conference times to scheduled simulations so that pediatric emergency medicine faculty, as well as rotating residents and other trainees (mainly from pediatrics and emergency medicine), would have a chance to participate and learn. Facilitators may review the simulation didactic (Appendix H) in preparation for leading the session. Additionally, less experienced learners could be given the didactic content provided in Appendix H in order to prime them for the simulation session. The five participating sites added different elements of realism depending on their capability. For instance, one site provided a dark liquid substance in a urine collection container to simulate dark urine that can be seen in rhabdomyolysis. Some sites used a low-fidelity mannequin, while others used a high-fidelity mannequin. At every site the patient was a teenager with serotonin syndrome.

### Equipment/Environment

The setting was an emergency department (ED) mental health room but could also be conducted in an ED room specifically designed for mental health patients, a regular ED room stripped of usual medical resuscitation equipment, a regular ED room, or a simulation lab. If participants start the scenario in a room intended for mental health patients, they may need to bring resuscitation equipment to the room or move the patient to a resuscitation room. One of our sites used a high-fidelity adolescent mannequin, which was able to sweat and make clonic-like movements. Another site used a low-fidelity manikin in the trauma bay of the pediatric ED to simulate a real environment. Other sites used a high-fidelity adolescent mannequin in a simulation lab that was set up identically to an ED resuscitation bay. If using a low-fidelity mannequin, vital signs may be provided verbally or by a simulator application for a phone or tablet, and the exam can be described verbally by a facilitator as learners examine the mannequin or learners can be told to ask the facilitator for the pertinent exam information. The environment preparation document (Appendix B) provided a complete list of suggested equipment. The patient was fully clothed and did not have IV access or attached monitors. The team was told that the patient was a 16-year-old male with a history of autism and behavioral issues brought in by his mom because of his agitation. Printouts or electronic versions of the electrocardiogram and chest radiograph were made available to trainees upon request or by verbal order. The simulation instructor provided verbal laboratory findings as requested/ordered by trainees as were reasonably available at the facility. The simulation instructor provided clinical changes and exam findings verbally throughout the scenario. If the learners started in a room with minimal equipment, they needed to move the patient and relevant materials to an appropriate resuscitation room or bring appropriate resuscitative equipment to the current patient room.

To demonstrate what clonus looks like if it cannot be achieved with the mannequin, an optional YouTube clip called *Ankle Clonus* can be played after the simulation.^28^

### Personnel

This simulation scenario was designed to accommodate from three to seven learners per session with a target audience of pediatric emergency medicine providers, including pediatric emergency medicine fellows and attendings, pediatric and emergency medicine nurses, and respiratory therapists. Learners were not required to complete any prerequisites before participating in this case; however, additional learning tools may be provided depending on the learners' background. The reference list contains recommended preparation materials (Appendix A). Participants should function in their normal roles (e.g., in an interdisciplinary simulation nurses should perform nursing roles and physicians should perform physician roles) for the simulation to be as realistic as possible. Required personnel included one simulation instructor/facilitator who also debriefed and provided educational instruction to trainees, as well as a simulation technologist who ensured a high-fidelity simulation. Additional individuals can play the actor roles (i.e., the patient's parent). If a second instructor is available, they may play the role of the parent to provide the patient's history. This instructor may also help with the debrief and education session following the scenario. If available, an actor may be considered instead of a mannequin. If a low-fidelity mannequin is utilized by an experienced simulation facilitator, the scenario may be run without a simulation specialist.

### Implementation

There was no specific prerequisite knowledge required by facilitators or learners, although most learners were expected to have some familiarity with the subject matter, as the simulation was targeted toward medical personnel who work in a pediatric emergency department. The scenario, as provided in detail in Appendix A, began with a parent bringing in an adolescent from home with altered mental status/agitation. Equipment and medications normally available in our clinical environment were available for the simulation participants (Appendix B). Additionally, Appendix C separated out critical actions for ease of facilitation. The triage nurse called in the team because the patient seemed very agitated. A confederate or the facilitator played the role of the parent who provided a brief history that the patient was found at home angry and aggressive, sweating, and unwilling to talk to the parent. Additional history was available at participant request. After a team member attached monitors, the vital signs listed in the simulation scenario were provided to the care team via a simulated monitor. The patient was agitated and required benzodiazepine treatment. The mannequin was then made to shake to simulate seizure and thus required more benzodiazepines. Ultimately, the patient developed respiratory failure secondary to seizure and benzodiazepine use, which necessitated an advanced airway. At the request of a participant, an electrocardiogram (Appendix D) was made available that was notable for sinus tachycardia, and a chest X-ray (Appendix E) was made available that was notable for an appropriately intubated patient. During treatment for serotonin syndrome, the patient developed rhabdomyolysis and required aggressive fluid hydration with at least two 20 cc/kg normal saline boluses and one and a half times the standard amount of intravenous fluids. Laboratory values for requested diagnostic testing were provided during the scenario (Appendix A). Appropriate sign-out of the patient to the pediatric intensive care unit was expected at the conclusion of the scenario. Appendix F provided a debriefing framework, Appendix G provided a glossary of terminology for teamwork and communication, Appendix H included a slide-based didactic, and Appendix I was the evaluation form for participants to complete after the case.

### Assessment

We described critical actions for the team to complete at each step of the simulation scenario according to the ideal care of the patient as well as in accordance with pediatric advanced life support (PALS) guidelines (Appendix C). The critical action checklist was based on the learning objectives and underwent a modified Delphi consisting three rounds of input by content experts, including a pediatric toxicology specialist (Suzan Mazor). There were prompts included in the simulation scenario provided for facilitators to help guide the team if an important step was missed or if facilitators felt the scenario was not going appropriately. Participants were able to evaluate the simulation case via Appendix I to assess alignment of their experience with our educational goals. Participants were asked to rate their agreement using a 5-point Likert scale (1 = *strongly disagree*, 2 = *disagree*, 3 = *neutral*, 4 = *agree*, 5 = *strongly agree*) with statements related to the fidelity and quality of the simulation, quality of the debrief, perceived confidence in knowledge, and skills and behaviors that were based on the learning objectives. The participant survey was created based on the learning objectives and evaluation forms used for other scenarios within the simulation curriculum and modified by pediatric emergency medicine and pediatric toxicology faculty for the details of this scenario. The survey was designed to include questions on scenario clinical content and the simulation learning environment, as well as open-ended questions for participants to list suggestions for further improvement as critical feedback to facilitators. This feedback form enabled us to tailor the scenario accordingly with subsequent iterations.

### Debriefing

After completing the simulation scenario, we decided the amount of time for debriefing and the education session with trainees and facilitators should be doubled. The debriefing materials (Appendix F) can be used to facilitate the debrief and discussion. Debriefing is a difficult skill and had a critical impact on the learning of the participants, thus making a structured and scripted debriefing method essential. Debriefings were conducted using the Promoting Excellence and Reflective Learning in Simulation (PEARLS) approach, which provided facilitators with a framework that they could tailor for individual learner groups.^29^ The PEARLS method uses a blended approach that ensures learning is active, collaborative, and learner centered through the use of three broad educational strategies: learner self-assessment, focused facilitation promoting reflection and understanding, and information provided via directive feedback. By using the PEARLS method, debriefers ranging from novice to experienced can guide learners through the debrief by selecting the debriefing strategy based on time constraints, rationale, content area, and learner insight. Debriefing discussion should cover teamwork and communication aspects of the scenario as well as medical management. The simulation session evaluation form (Appendix I) can be used to obtain feedback from the participants. The PowerPoint slides (Appendix H) are optional but can be used to help deliver educational content regarding serotonin syndrome, including recognition and management. We chose to use the supplemental PowerPoint slides after the simulation with more advanced learners, but they may instead be used prior to the simulation depending on learner needs.

## Results

We implemented the curriculum at five institutions with 57 participants, including 22 pediatric emergency medicine fellows, 30 emergency medicine residents, four pediatric emergency medicine attendings, and one medical student. All facilitators implementing this case were either currently pediatric emergency medicine trained fellows or faculty. Some faculty had additional training in simulation-based medical education.

The curriculum targeted Level 1 (i.e., reaction) of Kirkpatrick's Four-Level Training Evaluation Model.^5^ Evaluations (Appendix I) were filled out immediately following the debriefing. Additionally, during the debriefing participants were able to provide verbal feedback that not only informed subsequent iterations of the simulation curriculum, but also enabled facilitators to have an idea of the team's knowledge of serotonin syndrome. There were some elements of Level 2 (i.e., learning) of Kirkpatrick's model that became apparent during the debriefing when the case learning points were discussed. Additionally, formally trained simulation instructors often query learners during debriefings to state one piece of learning from the case. This simulation, however, was designed to be more formative than summative in terms of evaluating the team's knowledge and teamwork.

Learners filled out evaluations directly following participation in the simulation and debriefing. They rated questions (Table 1) on a 5-point Likert scale (1= *strongly disagree*, 2 = *disagree*, 3 = *neutral*, 4 = *agree*, 5 = *strongly agree*). Participants rated the simulation case as effective in teaching them how to both recognize (*M* = 4.9) and manage (*M* = 4.8) serotonin syndrome. Additionally, we asked participants to respond to two free-text questions. We organized their responses into several themes based on how participants would change how they do their jobs after completing the simulation (Table 2) and how the scenario could be improved (Table 3). Additionally, general comments regarding the simulation were collected from participants:

• “Awesome sim. Dark urine was a good touch.”
• “Great case! Thanks.”
• “Great case.”
• “Loved it!”
• “Great case! Relevant.”
• “Well thought out scenario.”
• “Wonderful sim! Brought together many isolated skills and thought processes, and encouraged systematic thinking.”
• “Good discussion after debrief and a good case to review.”
• “Great case! excellent- great review of tox/ddx wonderful sim!”

**Table 1.**
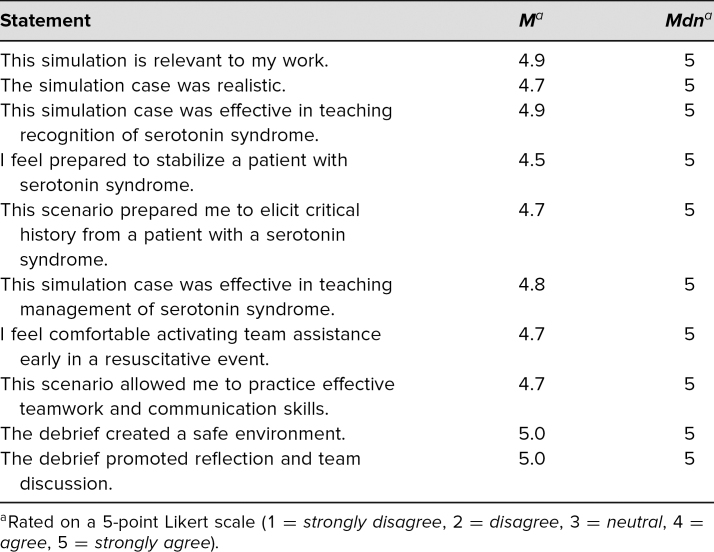
Participant Cumulative Evaluation Scores (*N* = 57)

**Table 2.**
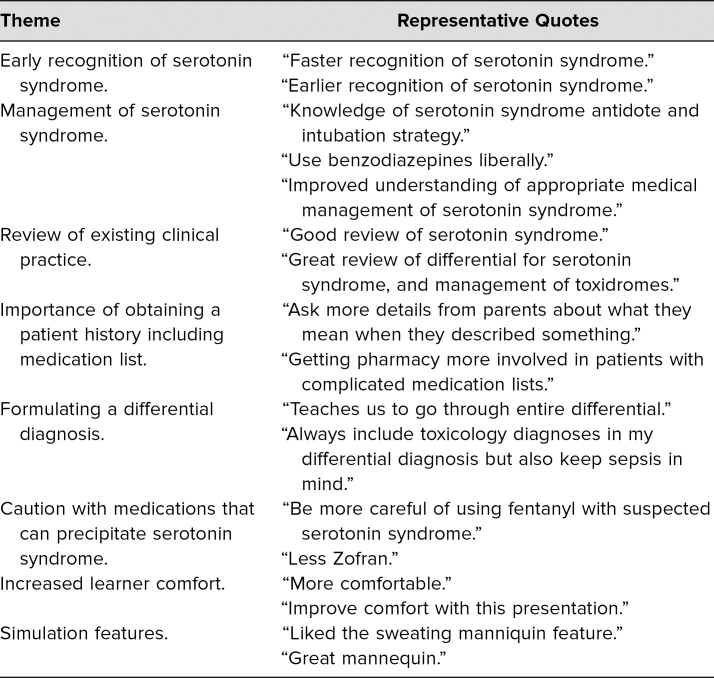
Themes Obtained From Participant Reponses to “Can You List/Describe One or More Ways This Simulation Session Will Change How You Do Your Job?”

**Table 3.**
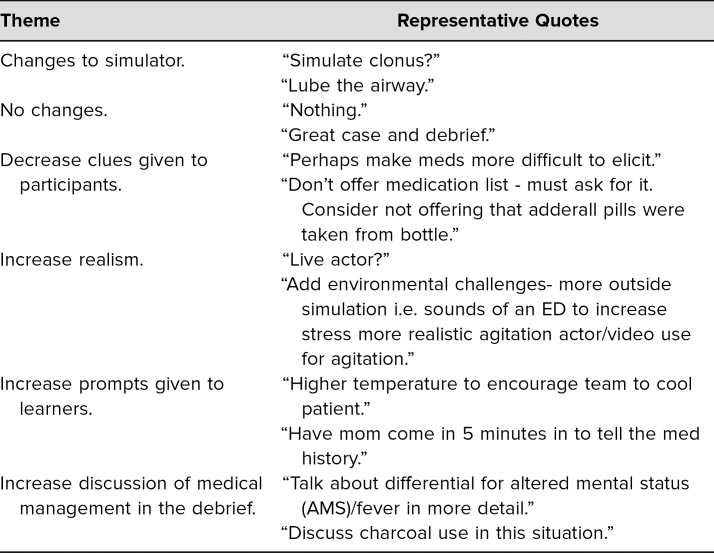
Themes Obtained From Participant Reponses to “How could we improve this scenario?”

## Discussion

This scenario was designed to be a comprehensive curriculum that provides guidance in teaching the recognition and management of serotonin syndrome with airway support and resultant rhabdomyolysis. Additionally, the simulation represents a typical presentation of serotonin syndrome and demonstrates an appropriate way for providers to gain familiarity with this illness. This simulation provides a low-frequency but high-risk scenario that is important for providers to rapidly recognize and treat appropriately in the pediatric and general emergency department settings. Given that many pediatric and emergency medicine providers see patients who may be on serotonin-related medications and patients with intentional overdose, it is crucial for providers to recognize and understand the management of serotonin syndrome so that they may promptly intervene with effective therapies.

Trialing this scenario was crucial to refining the curriculum material and adapting the scenario to learners of different levels. During the initial trial of this scenario, learners requested several lab values, as well as a chest X-ray, following intubation of the patient. These materials were not originally included in the materials for this case; however, following the initial trial we added these additional laboratory values and a chest X-ray. A limitation noted during a trial of this scenario was the inability to distinguish clonus versus seizure in the high-fidelity mannequin. This is a limitation of simulation even with high-fidelity mannequins, and thus the finding of clonus may be described or clarified by the simulation facilitator depending on the simulation technology available. During one trial of the scenario, the facilitator provided the learners with a dark brown urine sample, which was well received by learners, as they felt it aided in the diagnosis of rhabdomyolysis. We thus revised the optional materials to include a brown liquid as a urine sample that could be provided if requested by the learner. Additionally, several learners commented that the medications should be more difficult to elicit, or the participant playing the role of the parent should be introduced later in the scenario, so that the patient's past medical and medication history is not known up front. This may make it more difficult to identify serotonin syndrome, and thus may be considered with more advanced learners, such as pediatric emergency medicine fellows and attendings.

During a trial of this scenario with pediatric emergency medicine attendings and fellows, the debriefing discussion brought up the optimal rapid sequence intubation (RSI) medications for intubation. Given the underlying toxicity, we revised the intubation discussion to include the use of benzodiazepines as a sedative for RSI since benzodiazepines will concurrently treat the underlying serotonin syndrome as well.

One institution focused their training on emergency medicine residents and changed the scenario by decreasing the patient's age from 16 to 10 years old. This helped the emergency medicine residents understand the challenges of a pediatric patient. In addition, the parent actor was instructed to demonstrate an enhanced stress level and agitated state. The parent actor was also instructed to only provide the team with a list of medications if asked. The creatinine kinase and potassium levels were increased to better match the picture of severe serotonin syndrome.

### Limitations

Limitations in the evaluation of this educational activity included that the scenario was mainly tested on pediatric and emergency residents and pediatric emergency medicine faculty and fellows. The restrictions caused by using a mannequin, including the inability to simulate clonus, limited the realism of the scenario, but this may be mitigated by providing participants with a video clip of clonus. There may be aspects of this case that could be adapted to better serve other populations of health care workers, including physicians of other specialties, students, mid-level providers, and nurses, all of whom also care for this patient population. This curriculum was assessed primarily via Level 1 (i.e., reaction) of Kirkpatrick's model and does not delve into Level 2 (i.e., learning) of Kirkpatrick's model as much. To address Level 2 of Kirkpatrick's model, performance could be measured more formally if the simulation was repeated with the same group of learners multiple times over a prolonged time period; however, performing pre- and posttesting on all learners to assess performance/knowledge was not feasible. Additionally, we used a convenience sample of providers at the participating institutions, which may limit generalizability. However, given that the scenario was trialed at various institutions across the country and among varied populations of adult learners, the results are likely generalizable to the learner populations at other institutions. Translation of knowledge acquired from this session to actual clinical resuscitations was not measured by our evaluation tool, as this is an infrequently encountered patient presentation.

### Implications

The results from various sites informed further development and improvement of this simulation scenario and resulted in the inclusion of additional images, laboratory values, and clinical history. Our results highlighted that learners at various stages of training need different levels of prompting during the scenario in order to increase or decrease the challenge of diagnosing and/or managing the patient. Facilitators should account for their learners' unique needs and adapt the scenario accordingly. This scenario will continue to be used as part of the recurring pediatric emergency medicine simulation curriculum at the five participating institutions.

## Appendices

Simulation Case.docxSimulation Equipment Preparation.docxSimulation Critical Action Checklist.docxSimulation ECG.docxSimulation Intubated CXR.docxSimulation Debriefing Guide.docxSimulation Teamwork and Communication Glossary.docxSimulation Didactic.pptxSimulation Evaluation Form.docx
All appendices are peer reviewed as integral parts of the Original Publication.
