# Supplementary material for: Pediatric Emergency Medicine Didactics and Simulation (PEMDAS): Serotonin Syndrome
Source: MedEdPORTAL. 2020 Jul 28;16:10928. doi: 10.15766/mep_2374-8265.10928 (PMC7385927; doi:10.15766/mep_2374-8265.10928)
Supplement: Supplementary file 1 — Simulation Case.docxSimulation Equipment Preparation.docxSimulation Critical Action Checklist.docxSimulation ECG.docxSimulation Intubated CXR.docxSimulation Debriefing Guide.docxSimulation Teamwork and Communication Glossary.docxSimulation Didactic.pptxSimulation Evaluation Form.docx [file mep_2374-8265.10928-s001.zip › A. Simulation Case.docx]

| Appendix A: Serotonin Syndrome Simulation Case  Pediatric Emergency Medicine Didactics and Simulation: Serotonin Syndrome  Authors: | |
| --- | --- |
| PATIENT NAME: Jack  PATIENT AGE: 16 years old  PATIENT WEIGHT: 70 kg  CHIEF COMPLAINT: Agitation | |
|  | |
| Brief narrative description of case | Jack is a 16 year old male patient with a history of autism and behavioral issues brought in by his mom for acute agitation. Upon arrival to ED the patient is agitated, confused, tachycardic, hypertensive, diaphoretic, tremulous and hyperthermic. Anticipated initial interventions include performing a primary assessment, obtaining IV access, labs and an ECG.  The patient will have increasing agitation requiring treatment with benzodiazepines. Toxicology consultation should be considered, a differential diagnosis should be formulated, and a working diagnosis of serotonin syndrome chosen. The patient with have progression from agitation to clonus to altered mental status and seizure and will require frequent benzodiazepine dosing, intubation for airway stabilization, hyperthermia management with environmental cooling, intravenous fluids for rhabdomyolysis, and consideration of cyproheptadine for reversal. Throughout the case participants will need to frequently reassess the patient to determine the effectiveness of their interventions. Case is completed with admission to the PICU, participants may consult poison control, psychiatry. |
| Primary Learning Objectives | 1. Demonstrate ability to assess and emergently manage a pediatric patient with agitation, altered mental status and disability, including frequent reassessments. 2. Identify a possible toxicologic ingestion and formulate a differential, including serotonin syndrome. 3. Develop and execute a management plan for a patient with serotonin syndrome. 4. Identify and treat rhabdomyolysis.   5. Demonstrate effective team leadership, roles, and communication. |
| Critical Actions | Clinical State #1: Presentation   1. Complete primary and secondary patient assessments    1. Place patient on monitors    2. Obtain IV/IO access    3. Obtain diagnostic studies:       1. Labs: venous blood gas, electrolytes, toxicology screen, creatinine kinase, lactate       2. Electrocardiogram (ECG) 2. Collect focused history. 3. Develop differential diagnosis for agitated and altered patient 4. Identify serotonin syndrome. 5. Move patient to a resuscitation room, or alternatively bring appropriate resuscitation equipment to current patient room. 6. Begin treatment with benzodiazepines for agitation. 7. **Identify** possibility of co-ingestion.     Clinical State #2: Worsening Agitation and Seizure   1. Identify worsening agitation and seizure. 2. Treat seizure with benzodiazepines. 3. Initiate airway support, utilize airway adjuncts. 4. Perform intubation 5. Identify rhabdomyolysis and administer intravenous fluids.   Clinical State #3: Stabilization of Patient, Admission to Intensive Care Unit (ICU)   1. Transfer patient to ICU. 2. If diagnosis is confirmed, consider administering cyproheptadine prior to transfer. |
| Learner Preparation | - Optional Pre/post readings to supplement simulation: - Boyer E, Shannon M. The serotonin syndrome. N Engl J Med. 2005 Mar 17;352(11):1112-20.(<https://www.nejm.org/doi/full/10.1056/NEJMra041867>) - Chun, T, Mace S, Katz E, et al. Executive Summary: Evaluation and Management of Children and Adolescents With Acute Mental Health or Behavioral Problems. Part I: Common Clinical Challenges of Patients With Mental Health and/or Behavioral Emergencies. Pediatrics. 2016 Sep; 138(3): e1-e7. (https://pediatrics.aappublications.org/content/pediatrics/138/3/e20161571.full.pdf) |

| Initial Presentation | | | |
| --- | --- | --- | --- |
| Initial vital signs | Heart rate (HR) 125 Oxygen saturation (SpO2) 99% Blood Pressure (BP) 135/85 Respiratory Rate (RR) 25 Temperature (T) 39.5 degrees Celsius | | |
| Overall Appearance | 16 year old male brought in by parent. Patient sitting in bed, tremulous and agitated but not talking. | | |
| Actors and roles in the room at case start | Triage nurse has roomed agitated patient in mental health room (ideally a room stripped of usual medical supplies, such as oxygen, suction, etc), calls team into room to assess the patient.  Doctor #1: Team Leader  Doctor #2: Survey MD  Doctor #3: Airway Physician (if limited, this role can be combined with #2)  Nurse #1: Medication Administration Nurse (RN)  Nurse #2: Medication Preparation RN (optional)  Nurse #3: Documenting RN (optional)  Nurse #4: Circulating RN (optional)  All nurses and doctors can be at any level of training, depending on the audience the simulation is geared towards.  Instructor #1: Simulation instructor who will also act as debriefer  Instructor #2: If a 2^nd^ instructor is available, cast them as “parent,” available to answer questions and assist with debrief. If a 2^nd^ instructor is not available, the facilitator can play the role of parent as well. This instructor will also play role of PICU attending at the end of the scenario. | | |
| HPI | Jack is a 16 year old male with history of autism and behavioral issues brought in by mom for acute agitation.  On presentation, he is brought in by mother to the ED after mom found him uncontrollably agitated at home and uncommunicative with her. Per mom, she last saw him normal 1.5 hours prior to arrival when she denied his request to leave the house. When she went to check on him 1 hour later he appeared angry and aggressive and was noted to be "worked up," sweating, and unwilling to talk to her. This is not his first behavioral outburst requiring him to come to the ED, and with father's help they were able to load him into the car and bring him to the hospital.  SAMPLE history if asked:  Signs/symptoms- Sweaty, anxious, nonverbal young man  Allergies- none  Meds- citalopram, alprazolam, buspirone, melatonin, albuterol PRN  PMH: Autism, anxiety, depression, asthma. History of behavioral outbursts w/multiple presentations to ED. Mild autism and at baseline has normal verbal capacity and some social interaction.  Last intake: Unknown, mom presumes that he had lunch at school today  Events preceding- As above.  ROS: No recent illness  Social History: Lives with parents at home. Patient's meds are kept in bathroom upstairs. He also has access to acetaminophen, vitamins, and topical antibiotics. | | |
| Past Medical/Surgical History | Medications | Allergies | Family History |
| Autism, anxiety, depression, asthma. History of behavioral outbursts w/multiple presentations to ED. Mild autism and at baseline has normal verbal capacity and some social interaction. | Citalopram, alprazolam, buspirone, melatonin, albuterol PRN | None | None |
| Physical Examination (Primary survey) (Secondary survey)  Heart rate (HR) 125 Oxygen saturation (SpO2) 99% Blood Pressure (BP) 135/85 Respiratory Rate (RR) 25 Temperature (T) 39.5 degrees Celsius | | | |
| General | Agitated nonverbal patient. | | |
| HEENT | Patent airway, no signs of head trauma, pupils are 3mm-->2mm bilaterally, No hemotympanum, Nares clear without blood, oropharynx clear | | |
| Neck | Supple | | |
| Lungs | Clear to auscultation bilaterally, no stridor or wheezing | | |
| Cardiovascular | Tachycardic, 2+ peripheral pulses, capillary refill 2 sec | | |
| Abdomen | Abdomen soft, nontender, nondistended, normal bowel sounds, no masses | | |
| Neurological | Glasgow coma scale 12, follows commands, spontaneous opening of eyes, making sounds but no comprehensible words. Pupils 5mm and reactive. hyperreflexia LE>UE, clonus. | | |
| Skin | No rash or bruises. Warm, diaphoretic, flushed. | | |
| Genitourinary | Normal genitourinary exam | | |
| Psychiatric | Responds to exam by looking at examiner but not talking | | |

| Instructor Notes - Changes and CASE Branch Points | | |
| --- | --- | --- |
| Intervention / Time Point in Scenario | Change in Case | Additional Information |
| *Triage nurse notifies team that patient is very agitated, asks MD/team to come evaluate./ 0 minutes* | *Exam as above. Learners should establish team roles, assess ABCs, apply monitors, and obtain IV/IO access.* | *A: patent*  *B: clear to auscultation bilaterally*  *C: Tachycardic, 2+ pulses, CR 2 sec*  *D: GCS 12, looks at examiner and follows commands, spontaneous opening of eyes, making sounds but no comprehensible words. Pupils 5mm and reactive*  *E: abd soft, skin without abrasions, sweaty and warm* |
| *Temperature obtained* | *Learner should recognize hyperthermia and initiate cooling measures.*  *If antipyretic medication is given, patient temperature will not change.*  *If external cooling measures (fan, cool mist, cooling blanket, ice packs, etc.) are started, patient temperature will decrease to 38.5 C after 5 minutes.*  *If no external cooling measures are started, nurse says “should we do something about his temperature?”* |  |
| *Secondary survey, initial actions/ 3 minutes* | *Learner should:*  *- recognize tachycardia, hypertension and AMS.*  *-take SAMPLE history*  *-order labs including complete metabolic panel,Tox screen, tylenol and aspirin levels*  *-Order ECG*  *-Secondary survey*  *- Consider initiating a sepsis workup including lab work and antibiotics* | *GEN-agitated, tremulous*  *HEENT-pupils 5 mm and reactive, NCAT, TMs clear, dry mucous membranes*  *CV-tachycardic, no murmurs, cap refill < 2 seconds, 2+ pulses*  *LUNG-CTAB*  *AB-soft, NTND*  *NEURO/MSK-hyperreflexia LE>UE, clonus, arching back (Can provide overall neuro picture, but only provide info about reflexes and clonus if providers ask)*  *SKIN-sweaty, no rashes or lesion*  *ECG, CXR, lab results not yet available* |
| *Participant formulates a differential diagnosis./4 minutes* | *Learner should formulate a working differential diagnosis that includes serotonin syndrome and toxic ingestion.*  *If differential diagnosis is not formulated, nurse should prompt “what do you think is going on with this patient?”*  *If the differential does not include serotonin syndrome and toxic ingestion, can have the nurse prompt “mom was wondering if this could be due to his medications?”* |  |
| *IV access obtained/~5 minutes* | *After secondary survey and IV access, nurse says, "patient is becoming more agitated and tremulous!"* | *HR 140, Sat 97%, BP 170/100, RR 30, Temp 40*  *Exam as above when learner rechecks.*  *If a poison control consult is ordered, facilitator to state they will call back in 15 minutes.*  *If Tylenol is given there will be no change in patient temperature.*  *-Initiate cooling measures such as lowering room temperature, a cooling blanket, fans, mist or ice packs.* |
| *Participant requests to relocate patient* | *If in a mental health room, participants may recognize need to move patient into different medical room or resus room, or bring in additional equipment.*  *If participants do not move the patient or bring in additional equipment, nurse says “I don’t have all the equipment I need in this room.”* |  |
| *Participant requests labs* | *Lab results given only for those requested by learner:*  *If the participants do not request any labs, can have the nurse prompt when putting in the IV: “do you want me to collect any blood for labs?”* | *Venous Blood Gas (VBG)*  *ph=7.24 pCO2=34 pO2= 75 Bicarbonate = 20*  *Sodium 147, Potassium 4.7, Chloride 111, CO2 25, BUN 13, Creatinine 0.9, Glucose 132, Calcium total 10.2*  *Liver function tests (pending)*  *White blood cell count: 15.5, Hematocrit: 39.9, Hemoglobin: 13.7, Platelets: 196, Lactate 3.2*  *toxicology screen (serum or urine drug screen), acetaminophen and aspirin levels, and blood alcohol level (BAL) pending.* |
| *Participant requests electrocardiogram (ECG)* | *ECG provided* |  |
| *Dose of benzodiazepine is given* | *If a dose of benzodiazepine has been administered the patient’s tone and clonus will improve for a few minutes which can be prompted by the nurse saying “his arms and legs are much more relaxed now”* | *Dosing: lorazepam 2-4 mg IV or diazepam 5-10 mg IV can be given up to every 8- 10 minutes as needed*  *If a lorazepam dose of greater than 4 mg or diazepam dose of greater than 10 mg is given, then the patient becomes apneic and desaturates to 65% requiring bagging.* |
| *First seizure/10 minutes* | *Patient will have a generalized seizure at 10 minutes if no benzodiazepines have been administered.*  *If learner administered benzodiazepine as above, the patient improved briefly but will worsen again and require repeat dosing every few minutes.* | *If team does not give benzodiazepines, nurse can suggest giving IV lorazepam to treat agitation and seizure* |
| *Seizure management/12 minutes* | *Learner to support airway during and after seizure.*  *Seizure stops 1 minute after benzodiazepines given with brief improvement in agitation, clonus and return of respiratory drive.* | *With seizure, patient is tonic-clonic, becomes apneic and desaturates to 65%. With bagging patient saturating 99%, has clear breath sounds.*  *HR 150 SpO2 initially 65% but 99% with bagging BP 135/85 RR 9 without bagging T 39.5 degrees Celsius*  *After benzodiazepine given patient resumes breathing spontaneously with vitals same as above except RR at 18 and saturating 99% on room air without bagging.* |
| *Return of agitation and clonus/~15 minutes or 2-3 minutes after benzo given* | *3 minutes after resolution of seizure, patient will have increased tone and return of clonus. Worsening tone and clonus can be prompted by the nurse saying “his arms and legs are really stiff again”*  *Team must recognize need for frequent benzodiazepine dosing, continue to reassess ABCs.* | *Team may also consider benzodiazepine infusion.* |
| *Second seizure* | *Even if team has been administering benzos frequently, patient’s tone and clonus will worsen and proceed to seizure (or second seizure).* | *With seizure, patient is tonic-clonic, becomes apneic and desats to 65%. With bagging patient saturates 99%, has clear breath sounds.*  *HR 150 SpO2 initially 65% but 99% with bagging BP 135/85 RR 9 without bagging* |
| *Airway management* | *Team to manage airway during and after seizure. with bag-valve-mask.*  *Team to recognize persistent apnea requiring intubation after benzodiazepine dose given for second seizure. Team should intubate the patient or if less advanced learners, may request airway back up support. Seizure will stop after patient is intubated.* | *If chest x-ray (CXR) requested with intubation this can be provided.*  *Onceintubated, use benzodiazepine for sedative (will help with primary process)*  *HR 140 SpO2 initially 98% with bagging through endotracheal tube BP 135/85 RR 22 with bagging T 38 degrees Celsius*  *If end tidal CO2 requested, can provide team with reading of 36 or color change from purple to yellow.* |
| *Participant requests a Creatinine Kinase (CK)* | *Team to recognize concern for elevated creatinine kinase with clonus and seizures.* | *If CK requested on initial presentation, can result around 20 minutes into case. If requested after seizure can result quickly.*  *Provide CK lab result 9,890*  *K 6.0* |
| Recognition of rhabdomyolysis | *Recognize elevated CK and increased potassium as signs of rhabdomyolysis and administer fluid bolus, with consideration of bicarb* | *If team does not note the labs, PICU attending can arrive early and prompt diagnosis of rhabdomyolysis*  *Toxicology consult; discussion about charcoal if diagnosis made*  *Cyproheptadine started* |
| *Completion and signout to PICU/25 minutes* | *Team should identify that patient requires PICU admission and give signout to PICU attending, noting concerns for serotonin syndrome, rhabdomyolysis, and airway protection with sedation from benzodiazepines.*  *Sample sign out:*  *“This is a 16 year old male presenting with serotonin syndrome after likely overdose, intubated secondary to worsening seizures and use of benzodiazepines for agitation/seizure. He is also noted to have rhabdomyolysis likely secondary to seizure activity and so he is initiated on normal saline boluses x 2 and 1.5x maintenance intravenous fluids. “* | *Facilitator/Confederate can play role of PICU attending.* |

Ideal Scenario Flow

*Provide a detailed narrative description of the way this case should flow if participants perform in the ideal fashion.*

Participants are provided a brief scenario introduction and allotted two minutes to organize their team and assign roles. Participants enter the room to find an agitated patient. They immediately examine the patient, place patient on monitors and obtain intravenous access. Initial diagnostic testing includes laboratory studies and ECG. Teams recognize the possibility of serotonin syndrome and initiate treatment with benzodiazepines. The team continues to administer benzodiazepines, lorazepam 2-4 mg IV, diazepam 5-10 mg IV every 8-10 mins as needed, to treat the patient’s hypertonicity, clonus, and seizures. The team continuously reassesses the patient’s vital signs, airway, breathing and circulation. The team optimizes airway positioning, utilize airway adjuncts, and discusses the need for intubation. Teams may request toxicology or other specialty consultation, consultants will not be immediately available. Teams recognize and respond to patient’s hyperthermia by managing hypertonicity and clonus with benzodiazepines, consider other cooling measures (cooling blanket), or sedation/paralysis and endotracheal intubation.. Acetaminophen is not effective for treating hyperthermia in serotonin syndrome and should not be administered especially with high likelihood of co-ingestions. Teams diagnose and treat acute rhabdomyolysis with appropriate intravenous fluids. Team continues acute stabilization until facilitator arrives as confederate Intensive Care Unit physician and asks team leader for clinical summary statement.

Anticipated Management Mistakes

*Provide a list of management errors or difficulties that are commonly encountered when using this simulation case.*

*For example:*

1. *Failure to recognize underlying medical cause for patient’s acute agitation. Most of our learners expediently recognized the need for further workup, for teams who do not respond to the patient’s abnormal vital signs facilitators may act as a confederate nurse and draw participants attention to repeat abnormal vital signs.*
2. *Failure to recognize serotonin syndrome. Most participants quickly identified the possibility of serotonin syndrome and initiated treatment with benzodiazepines. If participants do not identify this, an embedded participant or the facilitator could play the role of a pharmacist and prompt the team about medications in case they do not recognize that one is an SSRI.*
3. *Failure to begin treatment with benzodiazepines. If participants do not initiate benzodiazepine therapy, facilitators may have the patient continue to seize or act as an embedded participant and suggest administration of benzodiazepines.*
4. *Failure to recognize and support the patient’s airway and breathing. Most learners reassessed airway, breathing and circulation frequently throughout the scenario and in response to interventions. If participants are not performing repeat assessments, facilitator may act as a confederate and call out a vital sign to prompt participants.*
5. *Failure to order appropriate labs, recognize and treat rhabdomyolysis. Many learners recognized potential for rhabdomyolysis, obtained appropriate labs and initiated treatment. If participants do not verbalize concern for rhabdomyolysis, obtain appropriate labs or initiate treatment, a facilitator may verbally prompt participants.*
