# Supplementary material for: Pediatric Emergency Medicine Didactics and Simulation (PEMDAS): Serotonin Syndrome
Source: MedEdPORTAL. 2020 Jul 28;16:10928. doi: 10.15766/mep_2374-8265.10928 (PMC7385927; doi:10.15766/mep_2374-8265.10928)
Supplement: Supplementary file 1 — Simulation Case.docxSimulation Equipment Preparation.docxSimulation Critical Action Checklist.docxSimulation ECG.docxSimulation Intubated CXR.docxSimulation Debriefing Guide.docxSimulation Teamwork and Communication Glossary.docxSimulation Didactic.pptxSimulation Evaluation Form.docx [file mep_2374-8265.10928-s001.zip › B. Simulation Equipment Preparation.docx]

**Appendix B: Serotonin Syndrome Simulation Environment Preparation**

Before each simulation, ensure the anticipated resuscitation equipment is available for the team’s use. The medications and equipment available should reflect what is actually available to participants in real practice. Not all medications or equipment are necessary for the simulation, as anticipated with ideal flow, but reflect what is often found in a pediatric emergency medicine department for the care of critically ill patients.

**Resources**

PALS reference cards, material

Patient Weight Estimator such as a Broselow tape

Pediatric Resuscitation Medication references (e.g.: Broselow tape, reference cards)

Documentation forms

Optional YouTube clip demonstrating clonus: <https://www.youtube.com/watch?v=XVlo1IB-DAc>

**Personal Protective Equipment Universal Precautions**

Staff gowns

Gloves

Mask and face shields

**Simulated Medications (consider having all or only a limited number of medications available; those bolded are expected to be used in this simulation)**

Acetaminophen

Adenosine

Amiodarone

Atropine

Ceftazidime

Ceftriaxone

**Cyproheptadine**

Epinephrine 1:10,000

Epinephrine 1:1,000

Etomidate (with appropriate dosing for intubation)

Fentanyl

Intralipid

Ketamine

Lidocaine

**Lorazepam**

**Midazolam**

Morphine

Norepinephrine

**Normal Saline/Lactated Ringers**

Ibuprofen

Procainamide

Rocuronium (with appropriate dosing for intubation)

Sodium bicarbonate

Succinylcholine

Vancomycin

**Equipment (consider having some or all equipment based on usual clinical environment)**

Simulator manikin in hospital gown or clothing, on bed with patient identification band

Monitor – NIBP, HR, RR, Oxygen saturation, temperature and ETCO2 monitor (if available)

Blood Pressure cuff, Heart Rate monitor leads, oxygen saturation probe, defibrillator cables and ETCO2 cannula (if available)

Oxygen hook-upsource - on wall or cylinder

Bag-valve-mask system, multiple size masks

Oxygen (O_2_ )– nasal cannula, mask - simple and/or non-rebreather

Suction device

Thermometer, temperature probe

Nasal, and oral airways, multiple sizes

Shoulder roll

Endotracheal tubes- 3.0, 3.5, 4.0, 4.5, 5.0, 6.0, 6.5, 7.0, 7.5 cuffed or uncuffed, stylets

Laryngoscope, Miller and Mac blades, multiple sizes

End-tidal CO2 colorimeter

Nasogastric tube(s)

Stethoscopes

IV/Angiocath, various sizes

IO needles, 2 sizes

Gauze, Tape

IV tubing/blood product tubing and filters

IV pumps, pressure bags/ blood product pumps

Syringes, multiple sizes

Bedside blood sample processors: glucose, electrolytes, gases

Specimen tubes

Code cart

CPR stool, backboard

Defibrillator / AED

Bedside ultrasound machine

High flow nasal cannula system and/or nasal CPAP system

Cooling measures i.e. fans, spray bottles with cool liquid, cool blanket, etc.
