# Supplementary material for: Pediatric Emergency Medicine Didactics and Simulation (PEMDAS): Serotonin Syndrome
Source: MedEdPORTAL. 2020 Jul 28;16:10928. doi: 10.15766/mep_2374-8265.10928 (PMC7385927; doi:10.15766/mep_2374-8265.10928)
Supplement: Supplementary file 1 — Simulation Case.docxSimulation Equipment Preparation.docxSimulation Critical Action Checklist.docxSimulation ECG.docxSimulation Intubated CXR.docxSimulation Debriefing Guide.docxSimulation Teamwork and Communication Glossary.docxSimulation Didactic.pptxSimulation Evaluation Form.docx [file mep_2374-8265.10928-s001.zip › C. Simulation Critical Action Checklist.docx]

**Appendix C: Critical Action Checklist**

Clinical State #1: Presentation

- Complete primary and secondary patient assessments
  - Place patient on monitors
  - Obtain IV/IO access
  - Obtain diagnostic studies:
    - Labs: venous blood gas, electrolytes, toxicology screen, creatinine kinase, lactate
    - Electrocardiogram (ECG)
- Collect focused history.
- Develop differential diagnosis for agitated and altered patient
- Recognize serotonin syndrome.
- Begin treatment with benzodiazepines for agitation
- Recognize possibility of co-ingestion.

Clinical State #2: Worsening Agitation and Seizure

- Identify worsening agitation and seizure.
- Treat seizure with benzodiazepines.
- Initiate airway support, utilize airway adjuncts.
- Perform intubation
- Identify rhabdomyolysis and administer intravenous fluids.
- Continue giving doses of benzodiazepines as needed for serotonin toxicity.

Clinical State #3: Stabilization of Patient, Admission to Intensive Care Unit (ICU)

- Continue giving doses of benzodiazepines as needed for serotonin toxicity.
- Transfer patient to ICU.
- If diagnosis is confirmed, consider administering cyproheptadine prior to transfer.
- Continue post-intubation sedation with either benzodiazepine or propofol drip
