# Supplementary material for: Pediatric Emergency Medicine Didactics and Simulation (PEMDAS): Serotonin Syndrome
Source: MedEdPORTAL. 2020 Jul 28;16:10928. doi: 10.15766/mep_2374-8265.10928 (PMC7385927; doi:10.15766/mep_2374-8265.10928)
Supplement: Supplementary file 1 — Simulation Case.docxSimulation Equipment Preparation.docxSimulation Critical Action Checklist.docxSimulation ECG.docxSimulation Intubated CXR.docxSimulation Debriefing Guide.docxSimulation Teamwork and Communication Glossary.docxSimulation Didactic.pptxSimulation Evaluation Form.docx [file mep_2374-8265.10928-s001.zip › F. Simulation Debriefing Guide.docx]

**Appendix F: Serotonin Syndrome Simulation Debriefing Materials**

**Debriefing Overview**

*We believe that reflective learning occurs in the DEBRIEF. It’s an opportunity for learners to reflect on their medical decision making, technical, teamwork and communication skills. The ultimate goal is to identify gaps and potential solutions to close those gaps, leading to improved patient safety and better quality care.*

**Framework for debriefing:**

- - - We model our debriefing after PEARLS ^1^which outlines 4 distinct phases of the debriefing process. Prior to starting the debrief, facilitators must set the stage for the debrief. Setting the stage allows learners to know this is a safe context for learning, discussing the basic assumptions of simulation-based learning and stating the general outline and goals of the debrief.
    - **Reactions phase**- opportunity for learners to express their emotional experience, where they may reveal key areas that are important to them
    - **Description phase-** opportunity for learners to summarize key events in the scenario to ensure that educators and learners are on the same page
    - **Analysis phase-** opportunity to explore the medical decisions, technical, teamwork and communication performance of the team
    - **Summary phase-** review of key take home points, led by learners or educator

General Debriefing Goals:

- Create a safe learning environment
  - Share a learning contract (PreBrief)
  - Normalize gaps in performance, if at all possible
- Ask open ended questions (avoid yes/no questions)
- Try to facilitate the **team’s** discussion (avoid lecturing)

1. **Reactions Phase**

There are different perspectives on emotions and debriefing. One perspective: until emotions are addressed, it’s difficult for adult learners to “move on” to an analysis of their performance and opportunities for improvement. Another perspective is that adult learners should process their emotions independently.

Our perspective is the first. If a group or team member is emotionally charged (e.g. sad, mad or frustrated), it’s usually difficult for the individual or the group to be actively engaged, receptive to feedback and able to promote learning, until the emotions are addressed.

An example: a medication error occurs. One team member may think it is all his/her fault. S/he may feel embarrassed, judged, etc. If he/she can verbalize this, other team members may offer different perspectives, which enable the team to process the error together, potentially identifying contributing systems issues. If the emotions aren’t addressed- 3 separate people may feel embarrassed, responsible and not engage in a discussion, failing to identify systems issues which led to the error.

What you might say:

- “How did that feel?”
- “How did that go?”
- “Initial reactions?”
- “How are the rest of you feeling?”

1. **Description phase**

Summary of key events to ensure that educator and participants are on the same page. Summary are best elicited from learners, but facilitators should provide guidance and focus if there is not a shared understanding of the case.

What you might say:

- “Could someone summarize the case so we are all on the same page?”
- “From your perspective, what were the main issues you dealt with?

1. **Analysis phase**

Promote reflection on performance (medical decision making, technical skills, teamwork and communication), identify opportunities for improvement. Facilitators may utilize both open ended questions allowing for learner self assessment as well as more focused facilitation and direct feedback to ensure learning objectives are met. Typically the authors utilize a combination of these strategies. The selection of debriefing strategies may be influenced by the time available for debriefing, the experience of the learners, and the experience of the facilitator.

What you might say:

- - - “Let’s talk more about the case.”
    - “What aspects did your team manage well? Why?”
    - “What could your team manage better next time? Why?”
    - “I want to spend a couple minutes talking about XXX. Can you tell me more about what was going on?”
    - I noticed you [*behavior*]…next time you may want to [*suggested behavior*]… because [*provide rationale*].

### **4) Summary phase**

Opportunity to review key learning points. Participants or educator can identify take home points. What you might say:

*Medical management/technical skills examples:*

- - - - “This was a scenario of a patient with serotonin syndrome who presented with altered mental status and agitation**, had a seizure, and then developed rhabdomyolysis. “**
      - **“Signs and symptoms of serotonin syndrome include altered mental status, agitation, autonomic instability, diaphoresis, clonus, neuromuscular hyperactivity, seizure, and myalgias.”**
      - “Focusing on the primary survey and having a systematic work up are critical for effective resuscitation.”
      - “The Evaluation and management of serotonin syndrome includes supportive care with attention to **seizure management and the possibility of developing rhabdomyolysis.**”
      - **“Management of serotonin syndrome includes: discontinuing serotonergic medications, treating with benzodiazepines and considering cyproheptadine, hydration, and minimizing hyperthermia.**”

*Teamwork/ communication examples:*

- - Recognize need for a full resuscitation team when a patient has altered mental status.
  - Designate leadership and team member roles to ensure coordinated team functioning.
  - Role assignment to specific individuals to avoid duplication/omission of tasks
  - Respect toward all team members is key to enable empowerment to speak up if patient safety issues arise.
  - Use brief or huddle to create a shared mental model for the working diagnosis and management plan.
  - Closed-loop communication is of paramount importance to ensure safe and adequate communication.

1. Eppich W and Cheng A. Promoting Excellence and Reflective Learning in Simulation (PEARLS): Development and Rationale for a Blended Approach to Health Care Simulation Debriefing. Sim in Healthcare. 2015:10 (2): 106-115.

**Debriefing Guide**

Below are examples of specific learning objective based statements & questions you may use to debrief the team during the analysis phase.

1. Demonstrate ability to assess and emergently manage a pediatric patient with agitation, altered mental status and disability, including frequent reassessments.
2. Identify a possible toxicologic ingestion and formulate a differential, including serotonin syndrome.
3. Develop and execute a management plan for a patient with serotonin syndrome.
4. Identify and treat rhabdomyolysis.
5. Demonstrate effective team leadership, roles, and communication.

| **Examples of debriefing for different learning objectives** | | |
| --- | --- | --- |
| **Perform a primary assessment** | | |
| Debriefer Script | Reference Material | Instructor Notes |
| “I noticed you [*quickly/took a while]* performed a complete primary assessment. This was [*great/could have been problematic]* since the primary assessment quickly identifies life threatening conditions.”   - “What [helped/hindered] you?” - “What could you do differently?” | - Primary assessment goals: Airway, Breathing, Circulation, Disability (e.g. pupils, Glasgow Coma Scale) and Exposure (remove clothing, temperature check and control) |  |

| **Recognizing relevant history for possible ingestion** | | |
| --- | --- | --- |
| Debriefer Script | Reference Material | Instructor Notes |
| “I noticed you *[quickly/took a while to]* identify that the history was concerning for possible ingestion. This was [*great/could be problematic]* since ingestions can lead to specific arrhythmias or neurologic complications.”   - “What [*helped/hindered*] you to [*recognize/treat*] it?” | - Historical elements potentially indicating ingestion: acute altered mental status, acute agitation, tremulous, diaphoretic, unable to speak, medications at home |  |

| **Develop and execute a management plan for a patient with serotonin syndrome** | | |
| --- | --- | --- |
| Debriefer Script | Reference Material | Instructor Notes |
| “I noticed you *[quickly/took a while]* to [*identify/treat*] serotonin syndrome. This was [*great/could be problematic]* since rapid recognition and treatment can help prevent complications.”   - “What [*helped/hindered*] you to [*recognize/treat*] it?” | Recognition of serotonin syndrome:   - Agitation, clonus, diaphoresis, hyperreflexia, hyperthermia, hypertonicity, tremor   Treatment of serotonin syndrome:   - Discontinue serotonergic agents - Benzodiazepines for symptomatic management, cyproheptadine - Control hyperthermia |  |
| **Identify and treat rhabdomyolysis** | | |
| Debriefer Script | Reference Material | Instructor Notes |
| “I noticed you *[quickly/took a while]* to [*recognize/initiate*] treatment for rhabdomyolysis. This was *[great/could have been even better]* because early [*recognition/treatment*], is critical to prevent complications*.”*   - “What [*helped/hindered*] you to [*recognize/treat*] rhabdomyolysis?” | Recognition of rhabdomyolysis   - Creatinine kinase level - Electrolyte monitoring   Initial treatment of rhabdomyolysis   - Aggressive fluid resuscitation - Electrolyte monitoring - Cardiac monitoring - at risk for arrhythmia |  |
|  | | |

| **Examples for debriefing teamwork learning objectives** | | | |
| --- | --- | --- | --- |
| **Roles and Responsibilities** | | | |
| Debriefer Script | Reference Material | | Instructor Notes |
| “From my perspective, it looked like you (*did/did not) have* clear team roles. I think this is (*great/concerning)* because clear team roles can help a team function smoothly, improving how quickly interventions take place and reducing errors.” | Team leader   - Clear direction, coordination, timely interventions - Stands at foot of patient with hands off of patient to see the big picture   Airway/Procedure MD   - Manage airway - Head of patient   Survey MD   - Primary, Secondary survey, pulses with CPR, reassess   Nursing roles   - Medication Prep (draw-up meds) - Medication Admin (give meds) - Documenting (time keeper) | |  |
| **Brief and Huddle** | | | |
| Debriefer Script | | Reference Material | Instructor Notes |
| “I noticed that your team *(did/didn’t/took a while to)* (*brief* *prior to the initial patient assessment/huddle after the initial evaluation).* I thought this (*was* *great/could have helped to*)facilitate patient care.”   - “What *(helped/hindered)* your team from (*briefing/huddling*)?” - “How did that impact your team?” - “What could your team have done differently?” - “How can you make sure that *(does/doesn’t*) happen again?” | | The goal of a brief/huddle is to create a shared mental model. Assure all team members know what the working diagnosis is, management priorities and next steps in care.   - Everyone on the team is responsible for making this happen. Anyone can ask for a brief/huddle. Brief/huddle is usually led by team leader. - If one team member doesn’t know what’s up or what’s next- s/he is probably not alone. |  |

| **Directed call out** | | | |
| --- | --- | --- | --- |
| Debriefer Script | Reference Material | | Instructor Notes |
| “I noticed that you (*did/didn’t/intermittently*) used (*peoples names/roles/eye contact*) when (*calling out orders/asking for assistance*). I thought this was (*great/could have been more directed*) in order to facilitate communication.”   - “What did you notice about orders/questions that were asked?” - “How did this impact your team?” | Directed call out. A tactical communication skill to assure that important orders/questions are specifically directed to one individual (rather than called out into the air).  Example:   - “Jennifer-What’s the saturation?” - “Kim- Give normal saline 500 mL” - “Team leader- she stopped responding to pain” | |  |
| **Closed loop communication/Check back** | | | |
| Debriefer Script | | Reference Material | Instructor Notes |
| “I noticed that you used closed-loop communication *(consistently/rarely)*. Closed-loop communication can be critical for catching errors and assuring that *(information/an order/a request)* is heard.”   - “Tell me about your communication loops” - “How did that impact your team?” - “Has anyone seen problems with this in a patient resuscitation? Can you tell us about that?” - “Has anyone seen closed loop communication prevent an error?” - “How could you do it differently next time?” | | Closed loop communication/check back is a strategy that requires verification of information. This enables the sender of the message to verify it has been heard and heard correctly. It enables the receiver to confirm what they heard is correct.   - Team leader “Float nurse, call for ECG” - Float nurse “calling technician for an ECG” - Team leader “correct” |  |

**Serotonin Syndrome Medical Management Evaluation/Debriefing Form**

This checklist identifies core medical management /technical skills. It’s hard to discuss more than 3 of these during one debriefing session. We recommend focusing on 2-3 of these issues.

**Performing a primary assessment □** Done Well  **□** Needs Work

Specific comments: _____________________________________________________________________

_____________________________________________________________________________________

*Discussion Points: What did you think of the timeliness/completeness of the ABCDE’s (airway, breathing, circulation, disability, exposure)? What could you do differently?*

**Recognize history concerning for possible ingestion □** Done Well **□** Needs Work

Specific comments: _____________________________________________________________________

_____________________________________________________________________________________

*Discussion Points: What historical elements were concerning for ingestion?*

**Develop a diagnostic and management plan for a patient with serotonin syndrome □** Done Well **□** Needs Work

Specific comments: _____________________________________________________________________

_____________________________________________________________________________________

*Discussion Points: What are the critical actions for patients with a suspected ingestion? Suspected serotonin syndrome?*

**Identify and treat rhabdomyolysis □** Done Well **□** Needs Work

Specific comments: _____________________________________________________________________

_____________________________________________________________________________________

*Discussion Points: What’s the emergent management for rhabdomyolysis?*

**Serotonin Syndrome Teamwork and Communication Evaluation**

This checklist identifies core teamwork and communication skills. It’s hard to discuss more than 3 of these during one debriefing session. We recommend focusing on 2-4 of these issues.

**Leader/Roles Identified & Maintained □** Done Well **□** Needs Work

Specific comments: ____________________________________________________________

____________________________________________________________________________

____________________________________________________________________________

*Discussion Points: What helped/hindered having clear leadership and roles?*

**Directed Call out □** Done Well **□** Needs Work

Specific comments: ____________________________________________________________

____________________________________________________________________________

____________________________________________________________________________

*Discussion Points: How were orders given- e.g. “Into the air” or directed at specific individuals? How did that impact you? How could they be delivered more effectively?*

**Check back/Closed loop communication □** Done Well **□** Needs Work

Specific comments: ____________________________________________________________

____________________________________________________________________________

____________________________________________________________________________

*Discussion Points: Describe closed loop communication.*

**Shared Mental Model □** Done Well **□** Needs Work

Specific comments: ____________________________________________________________

____________________________________________________________________________

____________________________________________________________________________

*Discussion Points: How did team members share information/working diagnosis/management plan ((brief/huddle)?*
