# Supplementary material for: Pediatric Emergency Medicine Didactics and Simulation (PEMDAS): Serotonin Syndrome
Source: MedEdPORTAL. 2020 Jul 28;16:10928. doi: 10.15766/mep_2374-8265.10928 (PMC7385927; doi:10.15766/mep_2374-8265.10928)
Supplement: Supplementary file 1 — Simulation Case.docxSimulation Equipment Preparation.docxSimulation Critical Action Checklist.docxSimulation ECG.docxSimulation Intubated CXR.docxSimulation Debriefing Guide.docxSimulation Teamwork and Communication Glossary.docxSimulation Didactic.pptxSimulation Evaluation Form.docx [file mep_2374-8265.10928-s001.zip › G. Simulation Teamwork and Communication Glossary.docx]

**Appendix G:** Serotonin Syndrome Simulation Teamwork and Communication (TeamSTEPPS) Glossary*

| **Term** | **Definition** |
| --- | --- |
| **Adaptability** | The ability to adjust strategies and alter a course of action in response to changing internal and external conditions. |
| **Call-Out** | A method to communicate critical information during an emergent event. Helps the team prepare for vital next steps in patient care. *(Example: “Airway status?” – “Airway clear”; “Breath sounds?” – “Breath sounds decreased on right”)* |
| **Check-Back or Closed-Loop Communication** | A communication strategy that requires a verification of information. The sender initiates the message; the receiver accepts it and restates the message. In return, the sender verifies that the re-statement of the original message is correct or amends if not. (*Example: “Give Benadryl 25 mg IV push” – “Benadryl 25 mg IV push” – “That’s correct”)* |
| **CUS** | Signal phrases that denote “I am **C**oncerned, I am **U**ncomfortable, This is a **S**afety Issue.” When spoken, all team members will understand clearly not only the issue, but the magnitude of the issue. |
| **Debrief** | Brief, informal information exchange session after an event designed to improve team performance and effectiveness. |
| **Pre-Brief** | Discussion prior to start of simulation/encounter to assign roles, establish expectations, anticipated outcomes, and contingencies. |
| **Shared Mental Model** | An organizing knowledge structure of relevant facts and relationships about a task or situation that is verbalized and held by team members. |
| **Situational Awareness** | The ability to identify, process, and comprehend the critical elements of information about what is happening to the team with regards to the mission. Knowing “What is going around you” and “What is likely to happen next” |

*Adapted from Reid J, Stone K. Pediatric emergency medicine simulation curriculum: hypovolemic shock. MedEdPORTAL. 2013;9:945
