# Supplementary material for: Pediatric Emergency Medicine Didactics and Simulation (PEMDAS): Serotonin Syndrome
Source: MedEdPORTAL. 2020 Jul 28;16:10928. doi: 10.15766/mep_2374-8265.10928 (PMC7385927; doi:10.15766/mep_2374-8265.10928)
Supplement: Supplementary file 1 — Simulation Case.docxSimulation Equipment Preparation.docxSimulation Critical Action Checklist.docxSimulation ECG.docxSimulation Intubated CXR.docxSimulation Debriefing Guide.docxSimulation Teamwork and Communication Glossary.docxSimulation Didactic.pptxSimulation Evaluation Form.docx [file mep_2374-8265.10928-s001.zip › H. Simulation Didactic.pptx]

## Slide 1
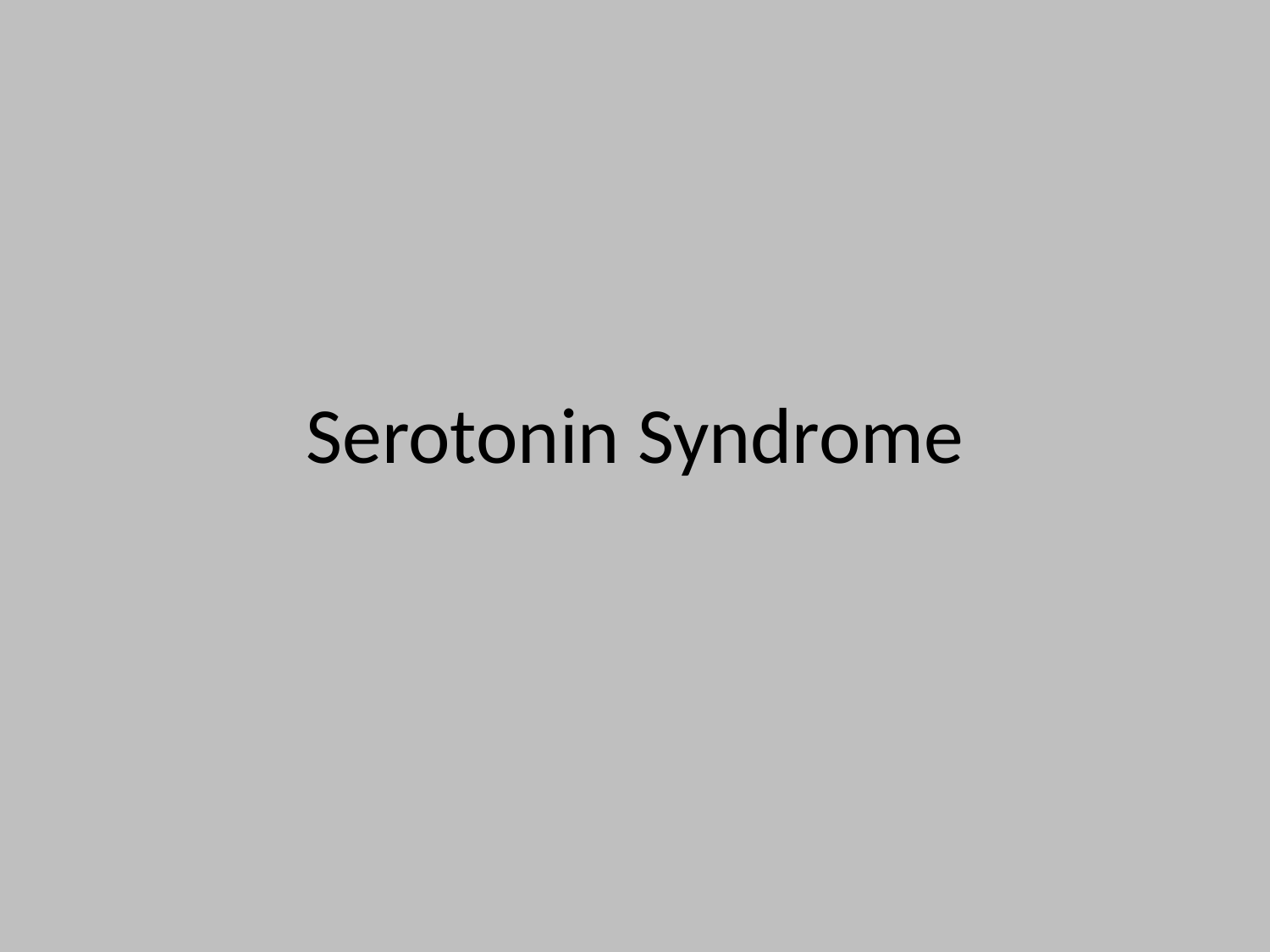

# Serotonin Syndrome

## Slide 2
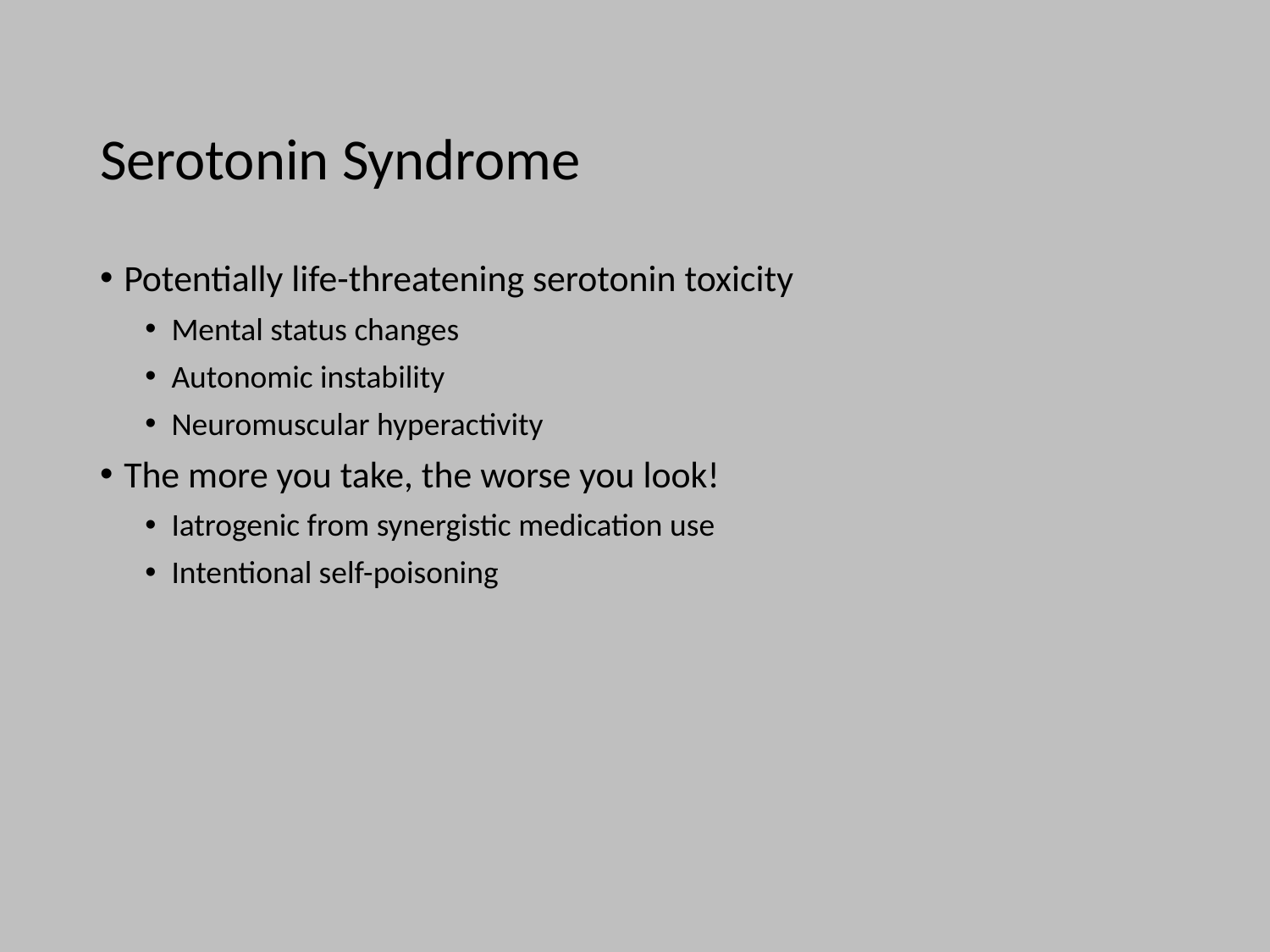

# Serotonin Syndrome
Potentially life-threatening serotonin toxicity
Mental status changes
Autonomic instability
Neuromuscular hyperactivity
The more you take, the worse you look!
Iatrogenic from synergistic medication use
Intentional self-poisoning

## Slide 3
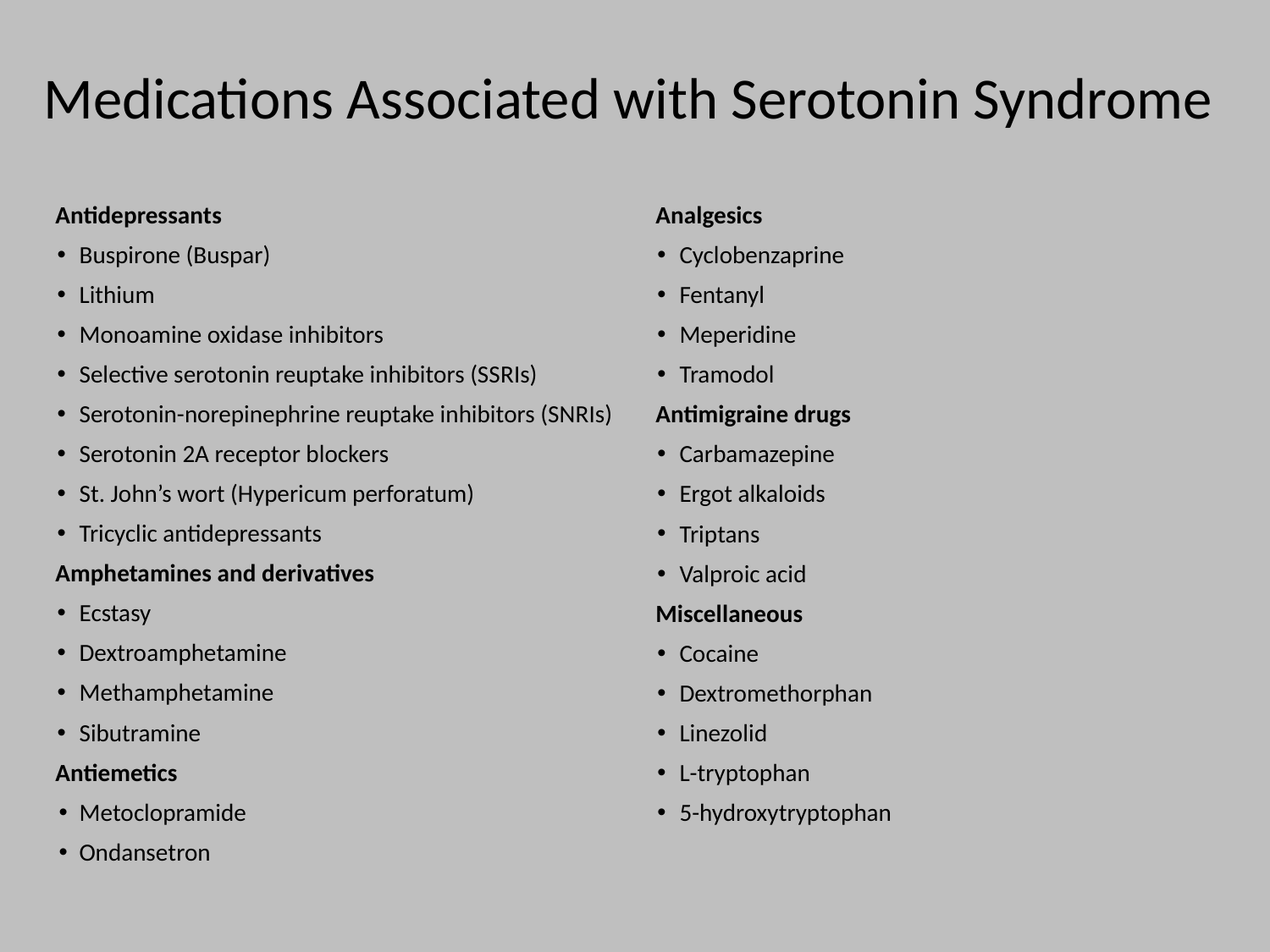

# Medications Associated with Serotonin Syndrome
Antidepressants
Buspirone (Buspar)
Lithium
Monoamine oxidase inhibitors
Selective serotonin reuptake inhibitors (SSRIs)
Serotonin-norepinephrine reuptake inhibitors (SNRIs)
Serotonin 2A receptor blockers
St. John’s wort (Hypericum perforatum)
Tricyclic antidepressants
Amphetamines and derivatives
Ecstasy
Dextroamphetamine
Methamphetamine
Sibutramine
Antiemetics
Metoclopramide
Ondansetron
Analgesics
Cyclobenzaprine
Fentanyl
Meperidine
Tramodol
Antimigraine drugs
Carbamazepine
Ergot alkaloids
Triptans
Valproic acid
Miscellaneous
Cocaine
Dextromethorphan
Linezolid
L-tryptophan
5-hydroxytryptophan

## Slide 4
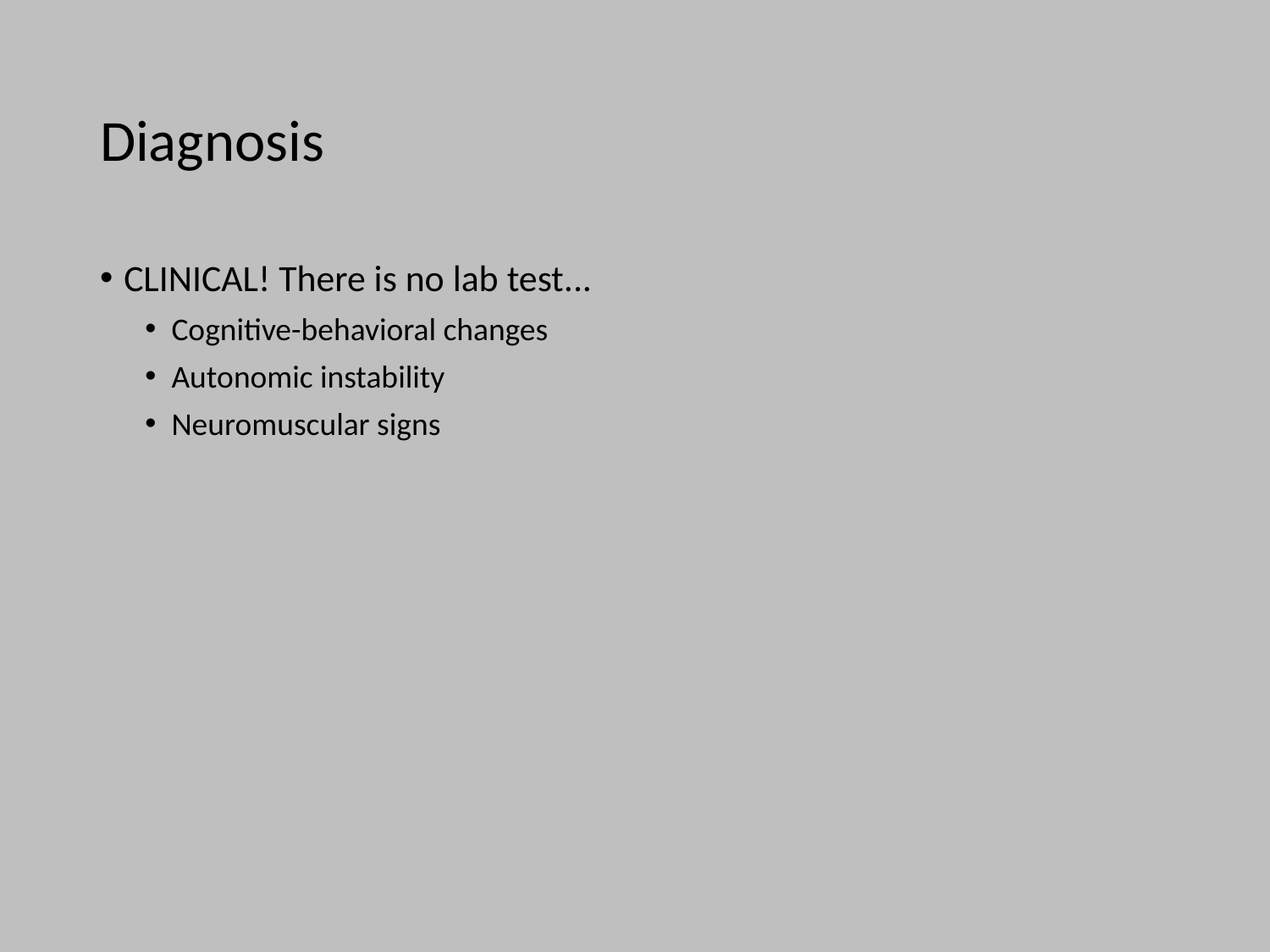

# Diagnosis
CLINICAL! There is no lab test...
Cognitive-behavioral changes
Autonomic instability
Neuromuscular signs

## Slide 5
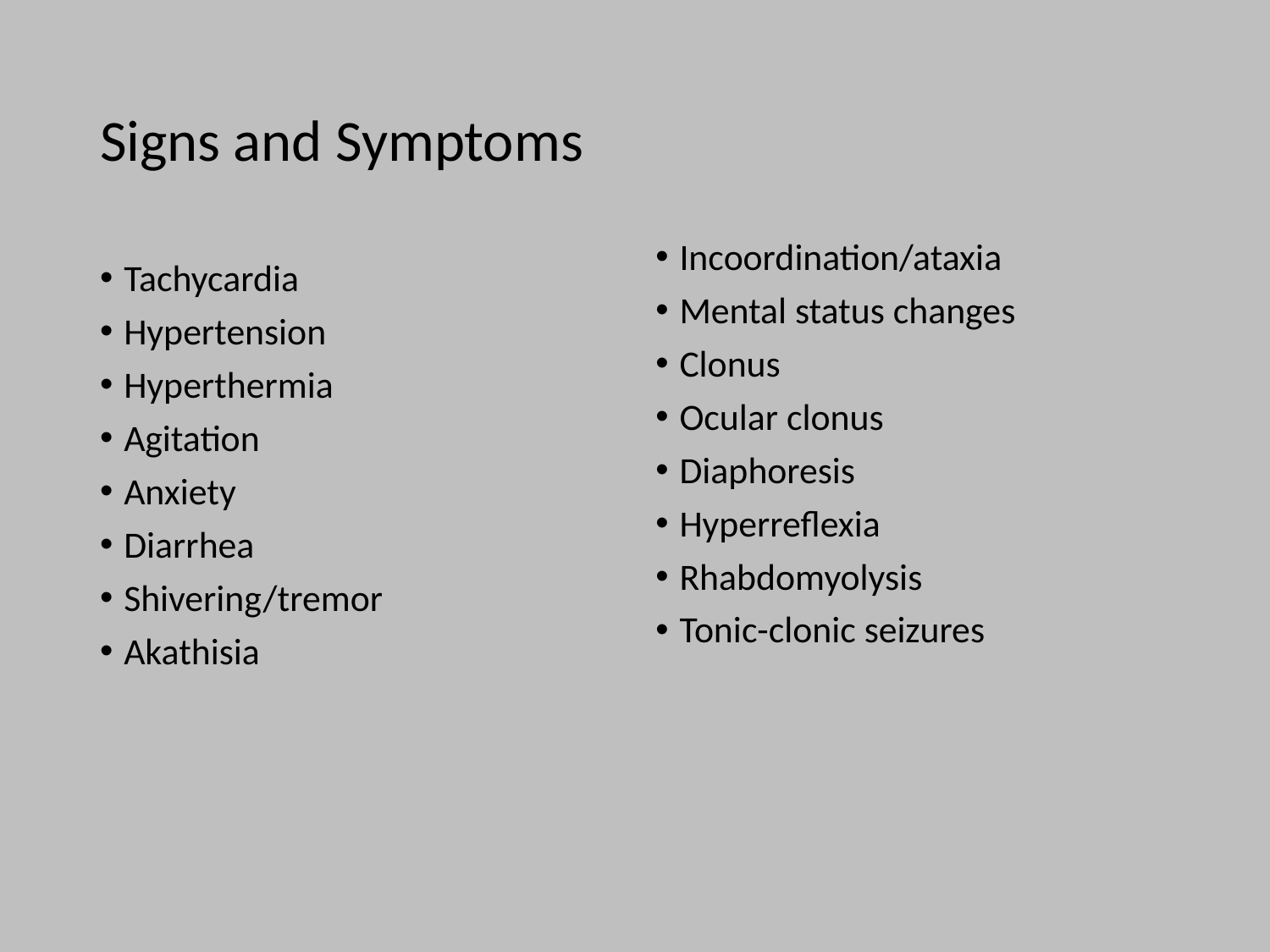

# Signs and Symptoms
Incoordination/ataxia
Mental status changes
Clonus
Ocular clonus
Diaphoresis
Hyperreflexia
Rhabdomyolysis
Tonic-clonic seizures
Tachycardia
Hypertension
Hyperthermia
Agitation
Anxiety
Diarrhea
Shivering/tremor
Akathisia

## Slide 6
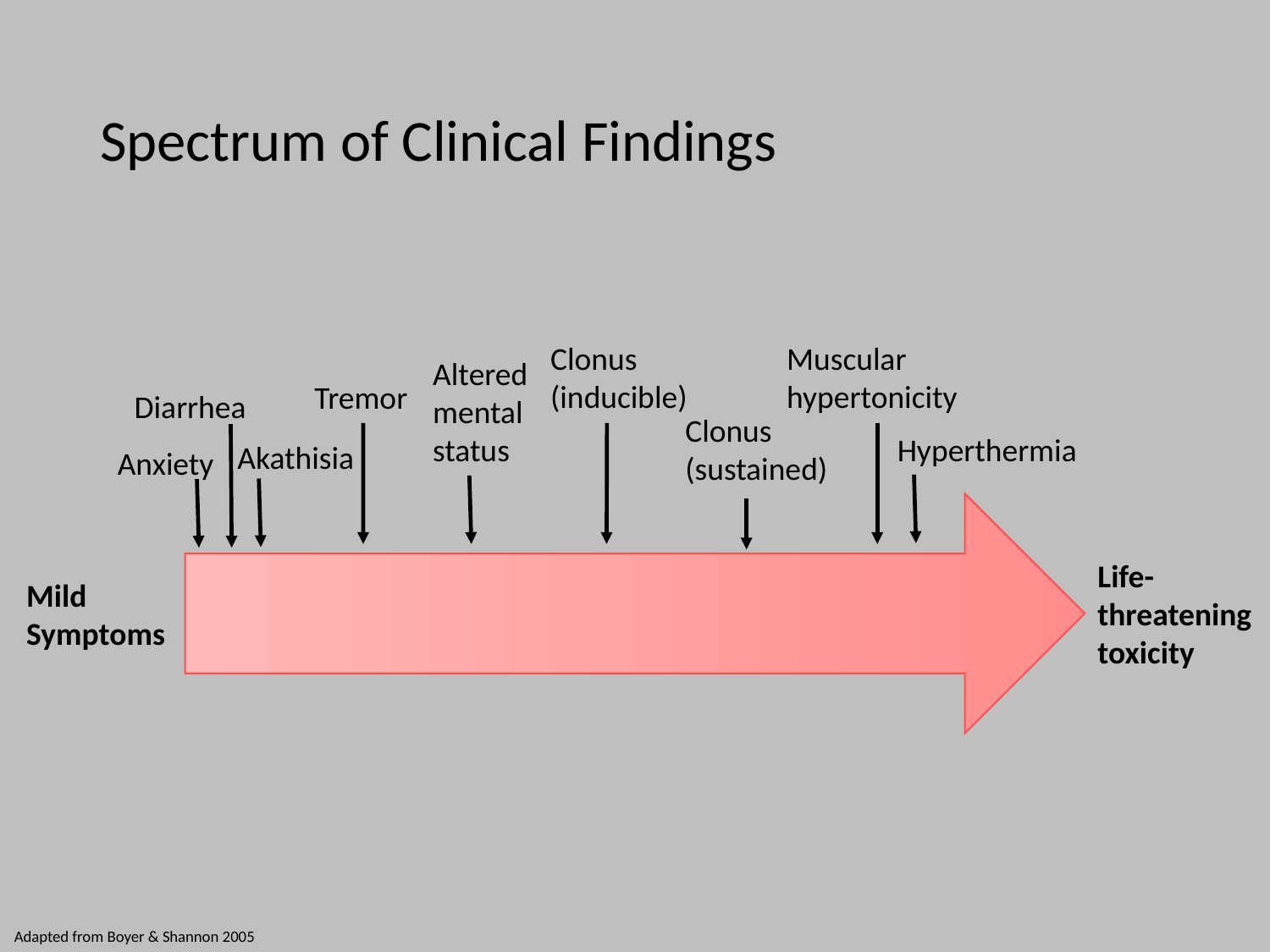

# Spectrum of Clinical Findings
Clonus (inducible)
Muscular hypertonicity
Altered mental status
Tremor
Diarrhea
Clonus (sustained)
Hyperthermia
Akathisia
Anxiety
Life-threatening toxicity
Mild Symptoms
Adapted from Boyer & Shannon 2005

## Slide 7
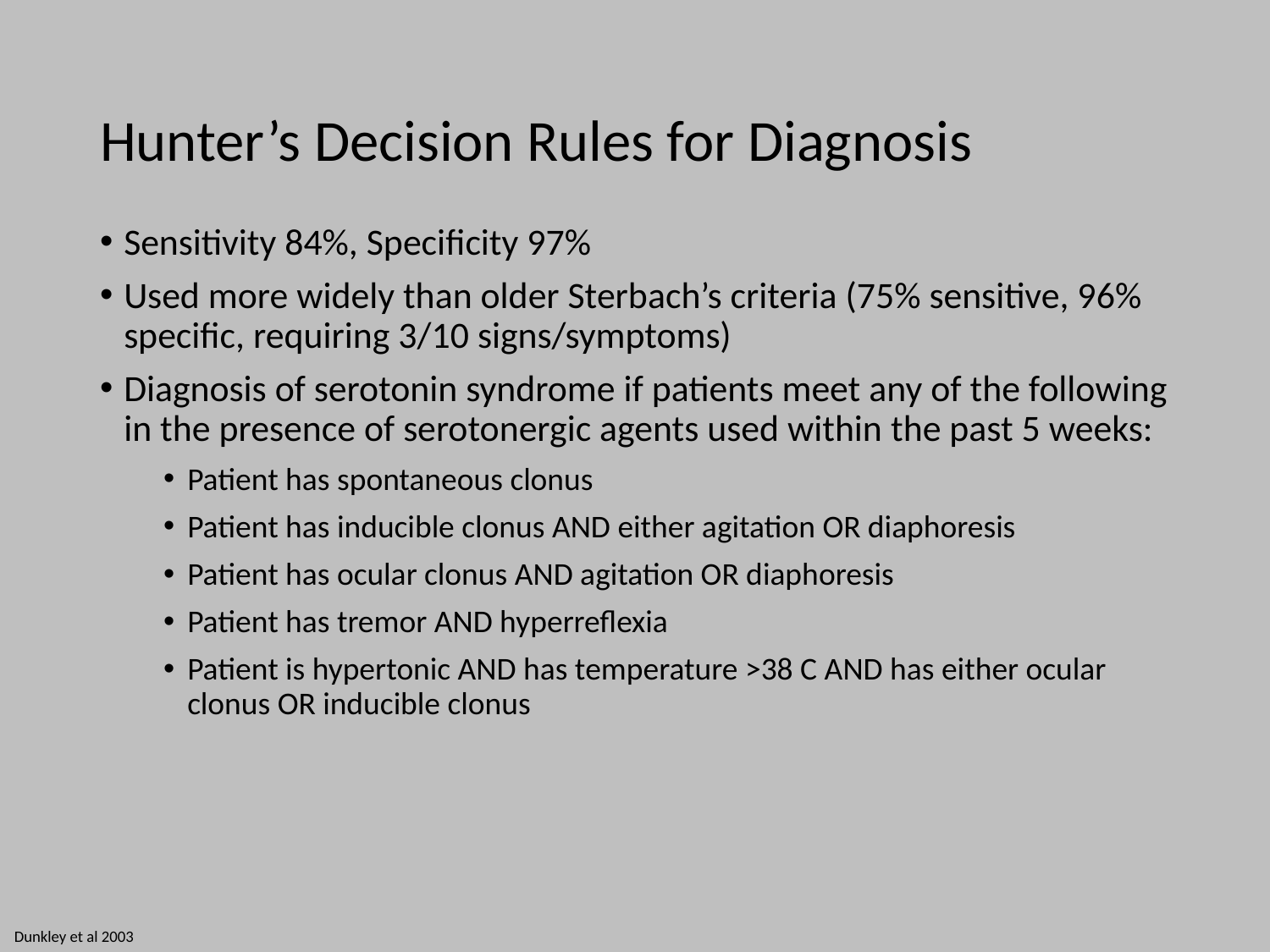

# Hunter’s Decision Rules for Diagnosis
Sensitivity 84%, Specificity 97%
Used more widely than older Sterbach’s criteria (75% sensitive, 96% specific, requiring 3/10 signs/symptoms)
Diagnosis of serotonin syndrome if patients meet any of the following in the presence of serotonergic agents used within the past 5 weeks:
Patient has spontaneous clonus
Patient has inducible clonus AND either agitation OR diaphoresis
Patient has ocular clonus AND agitation OR diaphoresis
Patient has tremor AND hyperreflexia
Patient is hypertonic AND has temperature >38 C AND has either ocular clonus OR inducible clonus
Dunkley et al 2003

## Slide 8
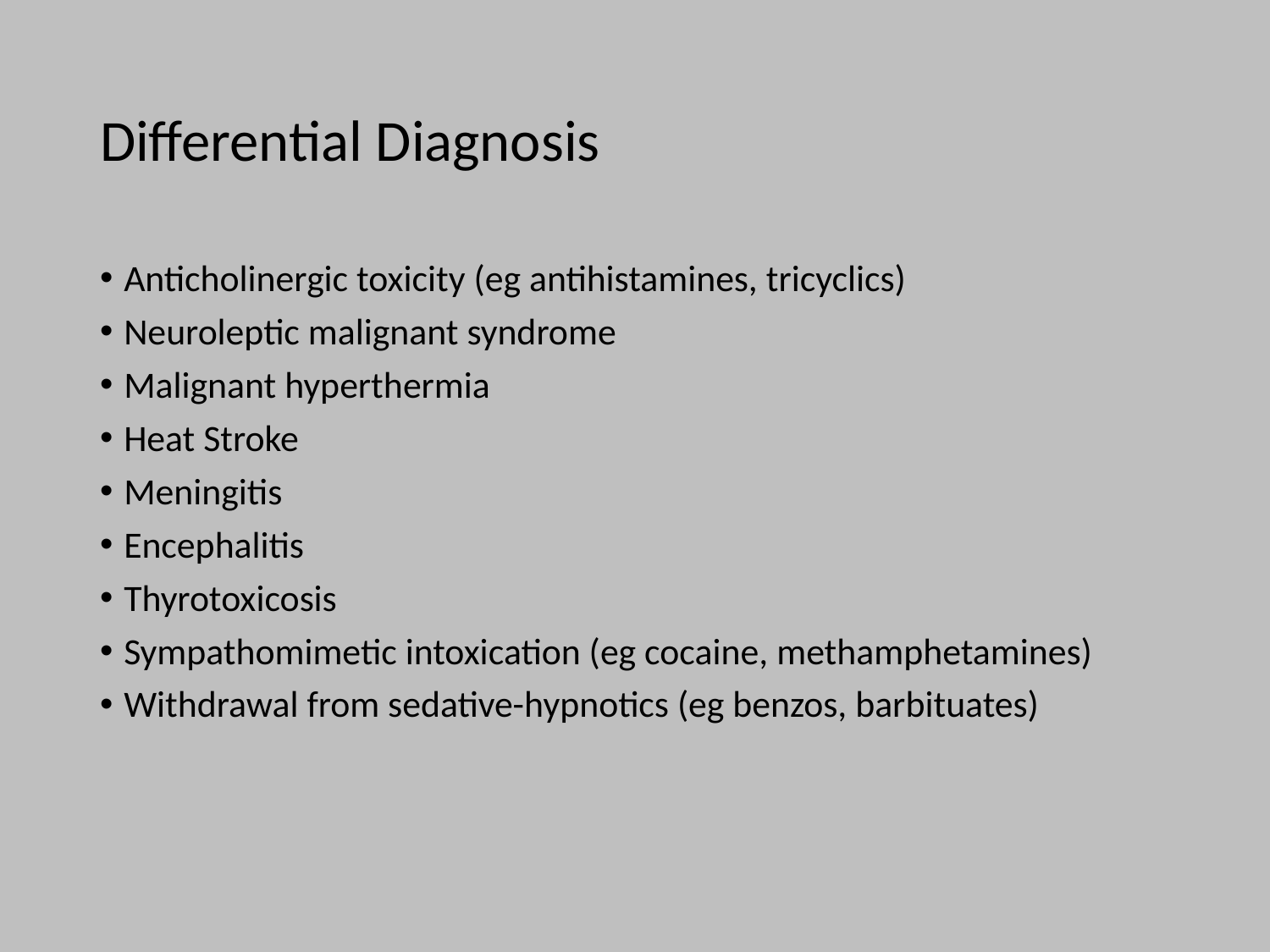

# Differential Diagnosis
Anticholinergic toxicity (eg antihistamines, tricyclics)
Neuroleptic malignant syndrome
Malignant hyperthermia
Heat Stroke
Meningitis
Encephalitis
Thyrotoxicosis
Sympathomimetic intoxication (eg cocaine, methamphetamines)
Withdrawal from sedative-hypnotics (eg benzos, barbituates)

## Slide 9
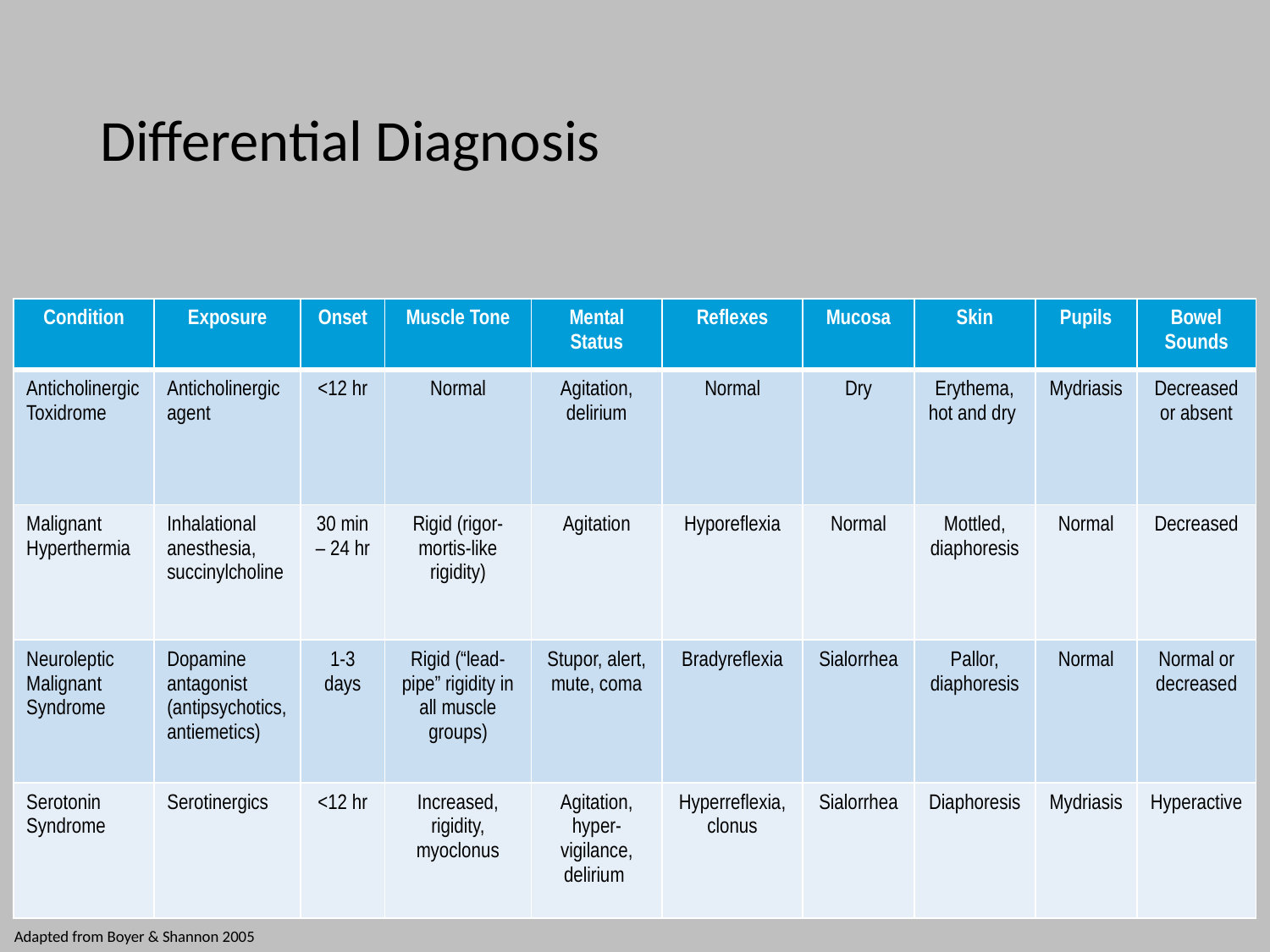

# Differential Diagnosis
| Condition | Exposure | Onset | Muscle Tone | Mental Status | Reflexes | Mucosa | Skin | Pupils | Bowel Sounds |
| --- | --- | --- | --- | --- | --- | --- | --- | --- | --- |
| Anticholinergic Toxidrome | Anticholinergic agent | <12 hr | Normal | Agitation, delirium | Normal | Dry | Erythema, hot and dry | Mydriasis | Decreased or absent |
| Malignant Hyperthermia | Inhalational anesthesia, succinylcholine | 30 min – 24 hr | Rigid (rigor-mortis-like rigidity) | Agitation | Hyporeflexia | Normal | Mottled, diaphoresis | Normal | Decreased |
| Neuroleptic Malignant Syndrome | Dopamine antagonist (antipsychotics, antiemetics) | 1-3 days | Rigid (“lead-pipe” rigidity in all muscle groups) | Stupor, alert, mute, coma | Bradyreflexia | Sialorrhea | Pallor, diaphoresis | Normal | Normal or decreased |
| Serotonin Syndrome | Serotinergics | <12 hr | Increased, rigidity, myoclonus | Agitation, hyper-vigilance, delirium | Hyperreflexia, clonus | Sialorrhea | Diaphoresis | Mydriasis | Hyperactive |
Adapted from Boyer & Shannon 2005

## Slide 10
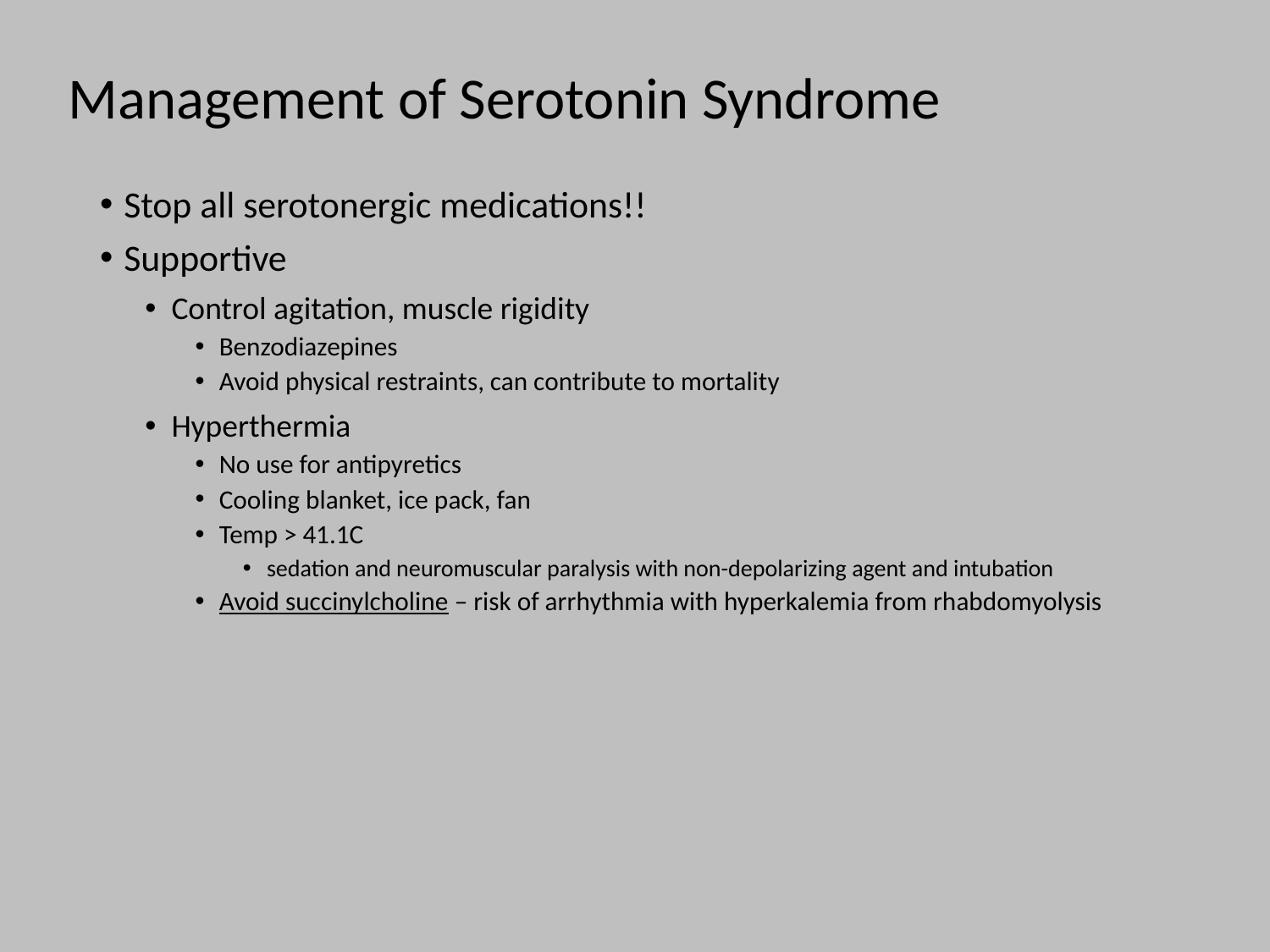

# Management of Serotonin Syndrome
Stop all serotonergic medications!!
Supportive
Control agitation, muscle rigidity
Benzodiazepines
Avoid physical restraints, can contribute to mortality
Hyperthermia
No use for antipyretics
Cooling blanket, ice pack, fan
Temp > 41.1C
sedation and neuromuscular paralysis with non-depolarizing agent and intubation
Avoid succinylcholine – risk of arrhythmia with hyperkalemia from rhabdomyolysis

## Slide 11
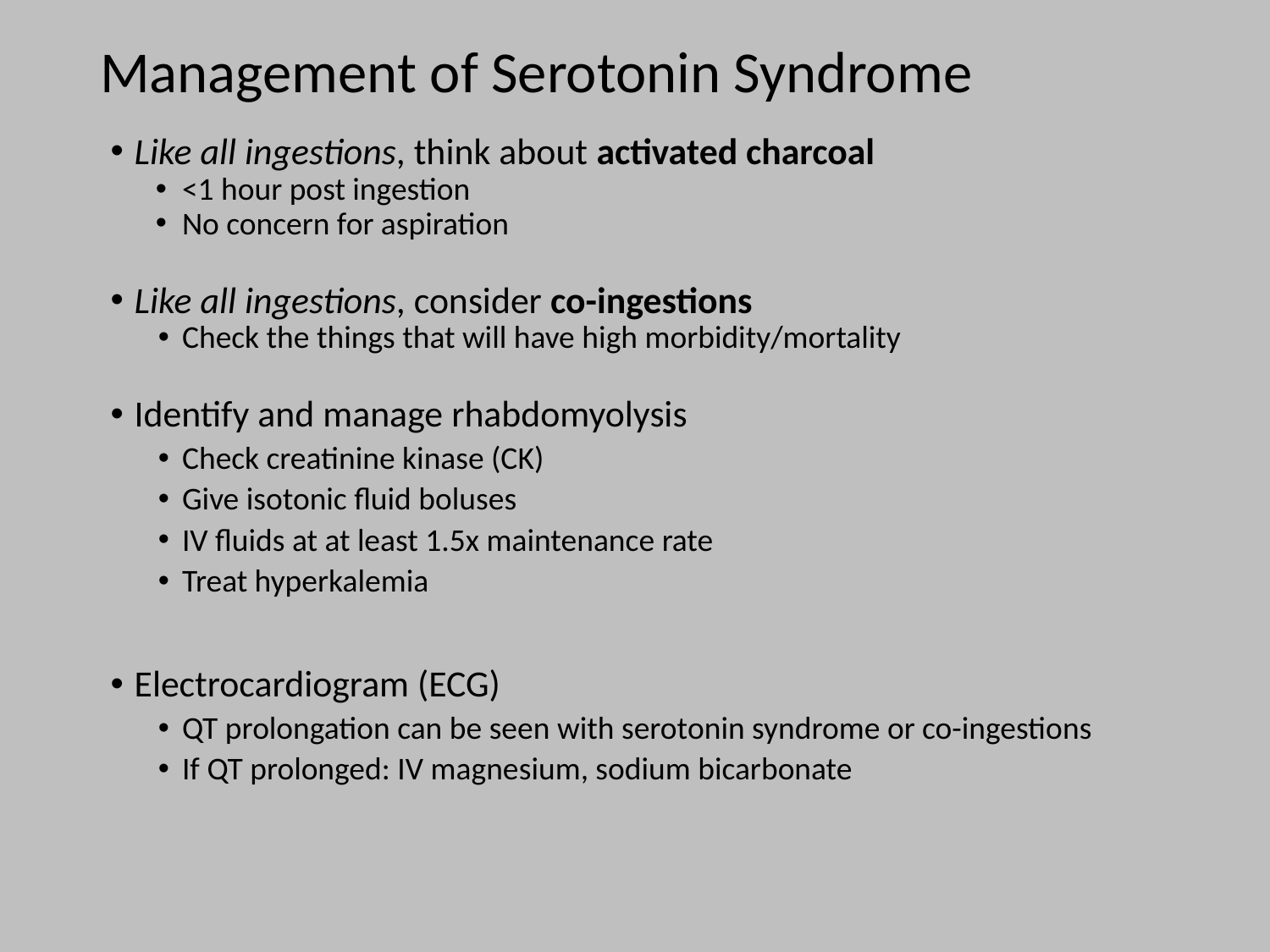

# Management of Serotonin Syndrome
Like all ingestions, think about activated charcoal
<1 hour post ingestion
No concern for aspiration
Like all ingestions, consider co-ingestions
Check the things that will have high morbidity/mortality
Identify and manage rhabdomyolysis
Check creatinine kinase (CK)
Give isotonic fluid boluses
IV fluids at at least 1.5x maintenance rate
Treat hyperkalemia
Electrocardiogram (ECG)
QT prolongation can be seen with serotonin syndrome or co-ingestions
If QT prolonged: IV magnesium, sodium bicarbonate

## Slide 12
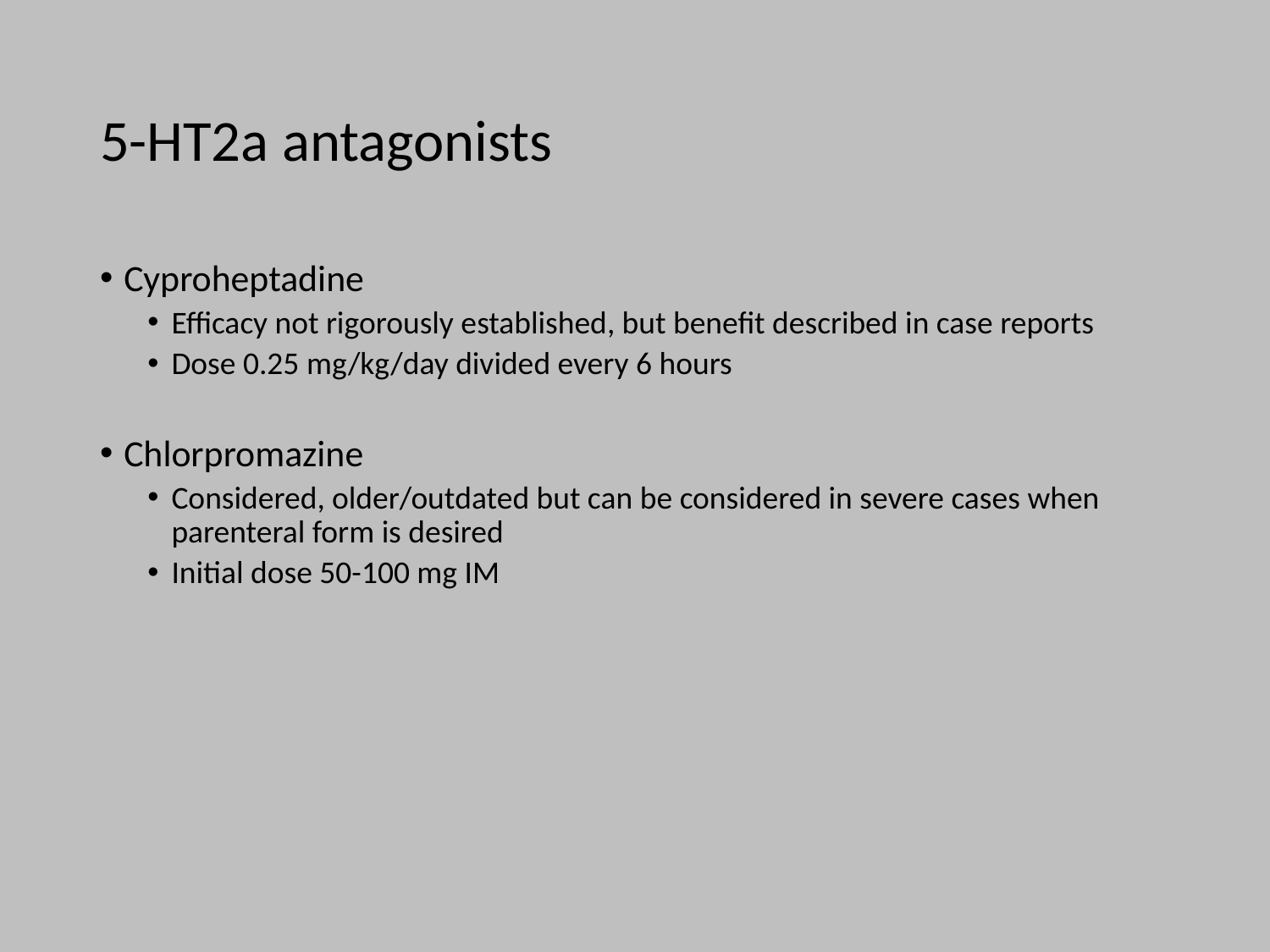

# 5-HT2a antagonists
Cyproheptadine
Efficacy not rigorously established, but benefit described in case reports
Dose 0.25 mg/kg/day divided every 6 hours
Chlorpromazine
Considered, older/outdated but can be considered in severe cases when parenteral form is desired
Initial dose 50-100 mg IM

## Slide 13
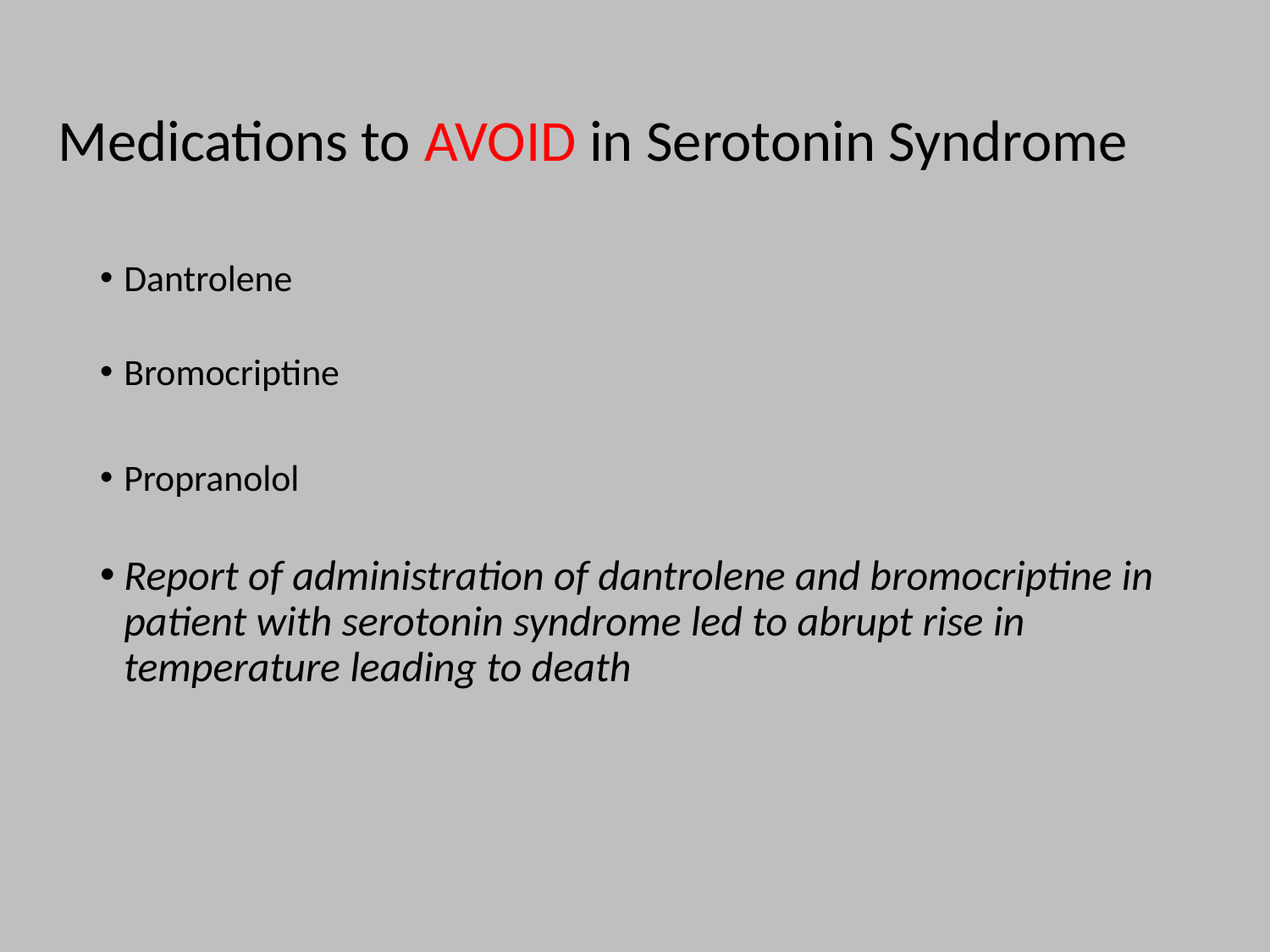

# Medications to AVOID in Serotonin Syndrome
Dantrolene
Bromocriptine
Propranolol
Report of administration of dantrolene and bromocriptine in patient with serotonin syndrome led to abrupt rise in temperature leading to death

## Slide 14
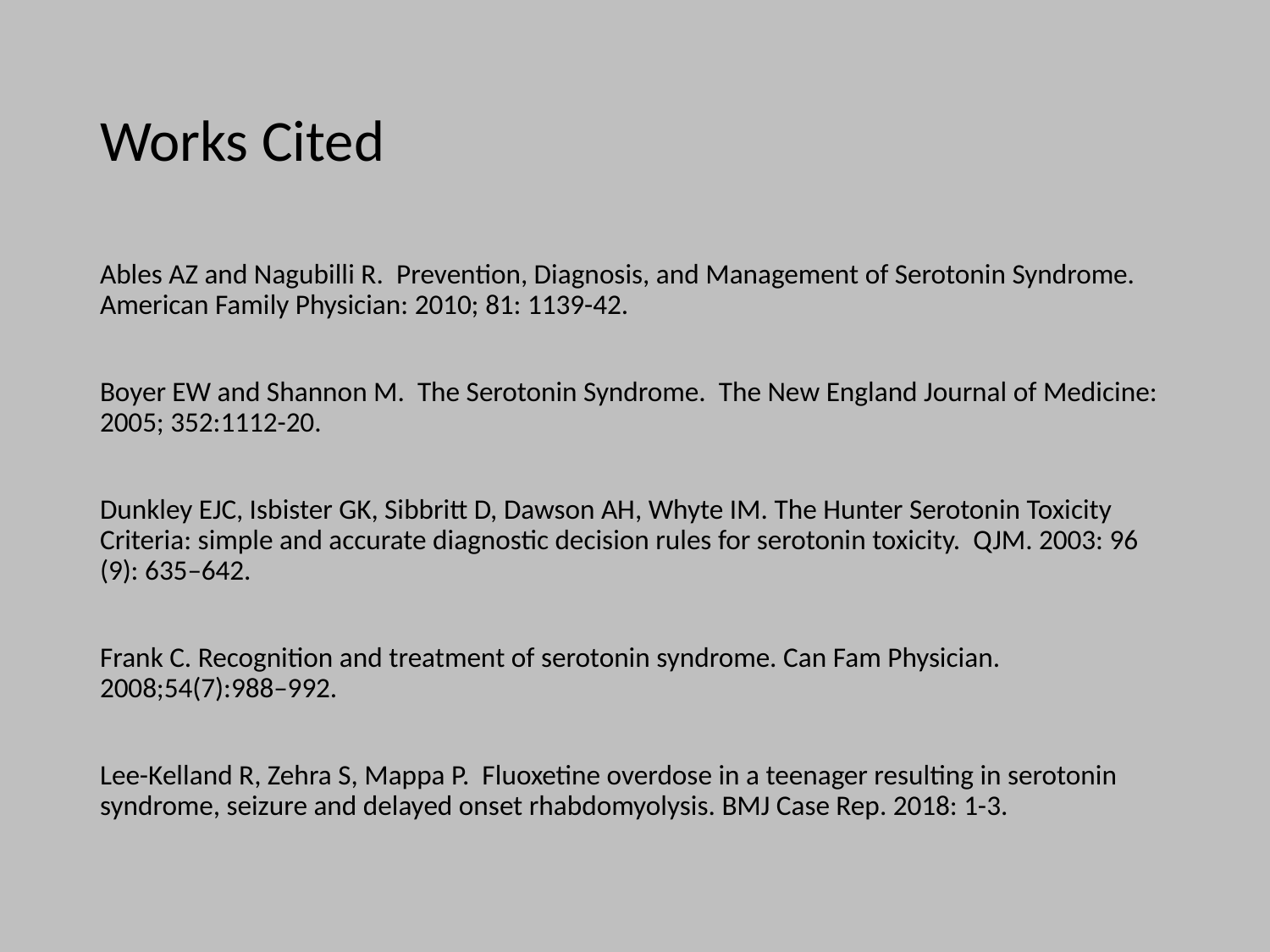

# Works Cited
Ables AZ and Nagubilli R. Prevention, Diagnosis, and Management of Serotonin Syndrome. American Family Physician: 2010; 81: 1139-42.
Boyer EW and Shannon M. The Serotonin Syndrome. The New England Journal of Medicine: 2005; 352:1112-20.
Dunkley EJC, Isbister GK, Sibbritt D, Dawson AH, Whyte IM. The Hunter Serotonin Toxicity Criteria: simple and accurate diagnostic decision rules for serotonin toxicity. QJM. 2003: 96 (9): 635–642.
Frank C. Recognition and treatment of serotonin syndrome. Can Fam Physician. 2008;54(7):988–992.
Lee-Kelland R, Zehra S, Mappa P. Fluoxetine overdose in a teenager resulting in serotonin syndrome, seizure and delayed onset rhabdomyolysis. BMJ Case Rep. 2018: 1-3.
