# Supplementary material for: Pediatric Emergency Medicine Didactics and Simulation (PEMDAS): Serotonin Syndrome
Source: MedEdPORTAL. 2020 Jul 28;16:10928. doi: 10.15766/mep_2374-8265.10928 (PMC7385927; doi:10.15766/mep_2374-8265.10928)
Supplement: Supplementary file 1 — Simulation Case.docxSimulation Equipment Preparation.docxSimulation Critical Action Checklist.docxSimulation ECG.docxSimulation Intubated CXR.docxSimulation Debriefing Guide.docxSimulation Teamwork and Communication Glossary.docxSimulation Didactic.pptxSimulation Evaluation Form.docx [file mep_2374-8265.10928-s001.zip › I. Simulation Evaluation Form.docx]

**Appendix F: Serotonin Syndrome Simulation Session Evaluation Form**

**Instructor(s):** ____________________________ **Date:**  _________________

**Circle One**: Medical Student/Resident/Fellow/Attending (Specialty:_______), Nurse, Other: _____

**Case Presented:** Serotonin Syndrome

|  | Strongly  Disagree | Disagree | Neutral | Agree | Strongly  Agree |
| --- | --- | --- | --- | --- | --- |
| 1. This simulation case provided is relevant to my work. | 1 | 2 | 3 | 4 | 5 |
| 1. The simulation case was realistic. | 1 | 2 | 3 | 4 | 5 |
| 1. This simulation case was effective in teaching recognition of serotonin syndrome. | 1 | 2 | 3 | 4 | 5 |
| 1. I feel prepared to stabilize a patient with serotonin syndrome. | 1 | 2 | 3 | 4 | 5 |
| 1. This scenario prepared me to elicit critical history from a patient with a serotonin syndrome. | 1 | 2 | 3 | 4 | 5 |
| 1. This simulation case was effective in teaching management of serotonin syndrome. | 1 | 2 | 3 | 4 | 5 |
| 1. I feel comfortable activating team assistance early in a resuscitative event. | 1 | 2 | 3 | 4 | 5 |
| 1. This scenario allowed me to practice effective teamwork and communication skills. | 1 | 2 | 3 | 4 | 5 |
| 1. The debrief created a safe environment. | 1 | 2 | 3 | 4 | 5 |
| 1. The debrief promoted reflection and team discussion. | 1 | 2 | 3 | 4 | 5 |

Can you list/describe 1 or more ways this simulation session will change how you do your job

How can we improve this scenario?

Comments:
