# Supplementary figures and images for: Pediatric Emergency Medicine Didactics and Simulation (PEMDAS): Serotonin Syndrome
Source: MedEdPORTAL. 2020 Jul 28;16:10928. doi: 10.15766/mep_2374-8265.10928 (PMC7385927; doi:10.15766/mep_2374-8265.10928)

**Appendix D**: Electrocardiogram (ECG):


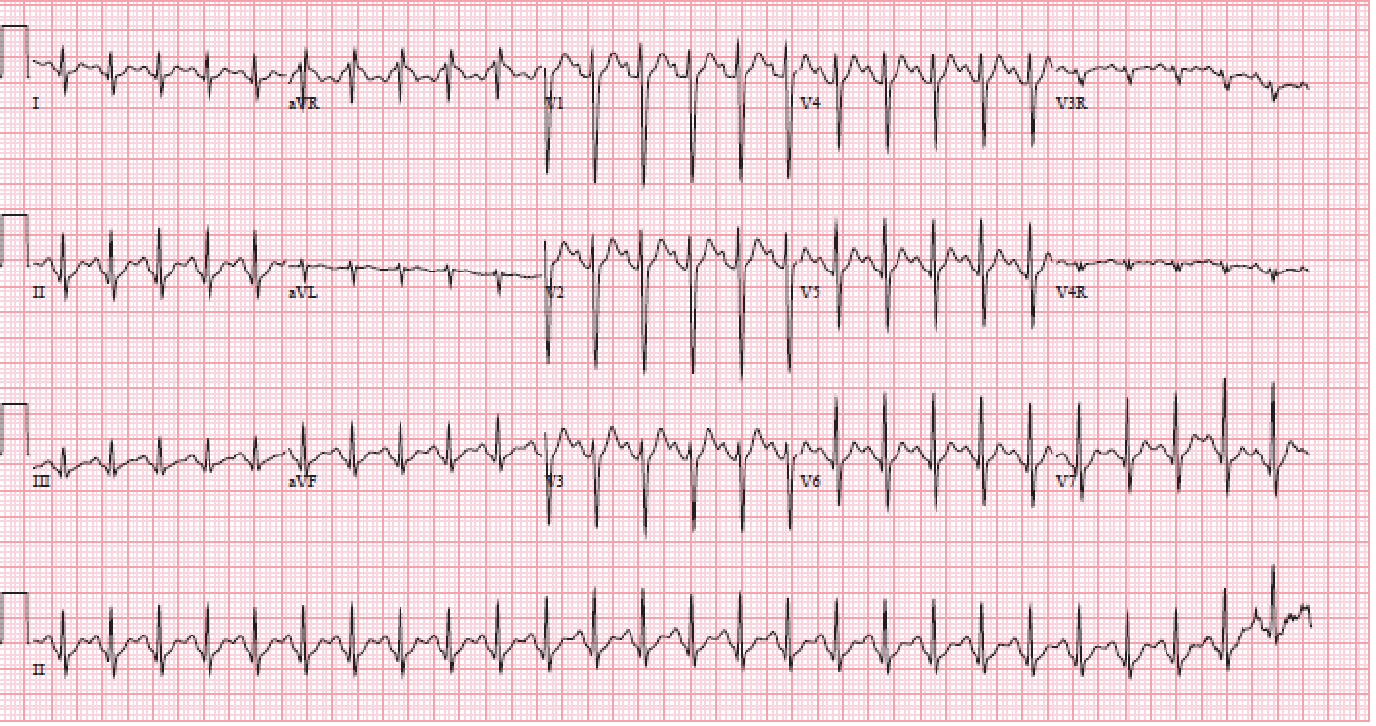


*Author owned: Dr. Corinne Shubin*

Supplement: Supplementary file 1 — Simulation Case.docxSimulation Equipment Preparation.docxSimulation Critical Action Checklist.docxSimulation ECG.docxSimulation Intubated CXR.docxSimulation Debriefing Guide.docxSimulation Teamwork and Communication Glossary.docxSimulation Didactic.pptxSimulation Evaluation Form.docx [file mep_2374-8265.10928-s001.zip › D. Simulation ECG.docx]

**Appendix E**: Intubated Chest X-Ray (CXR)


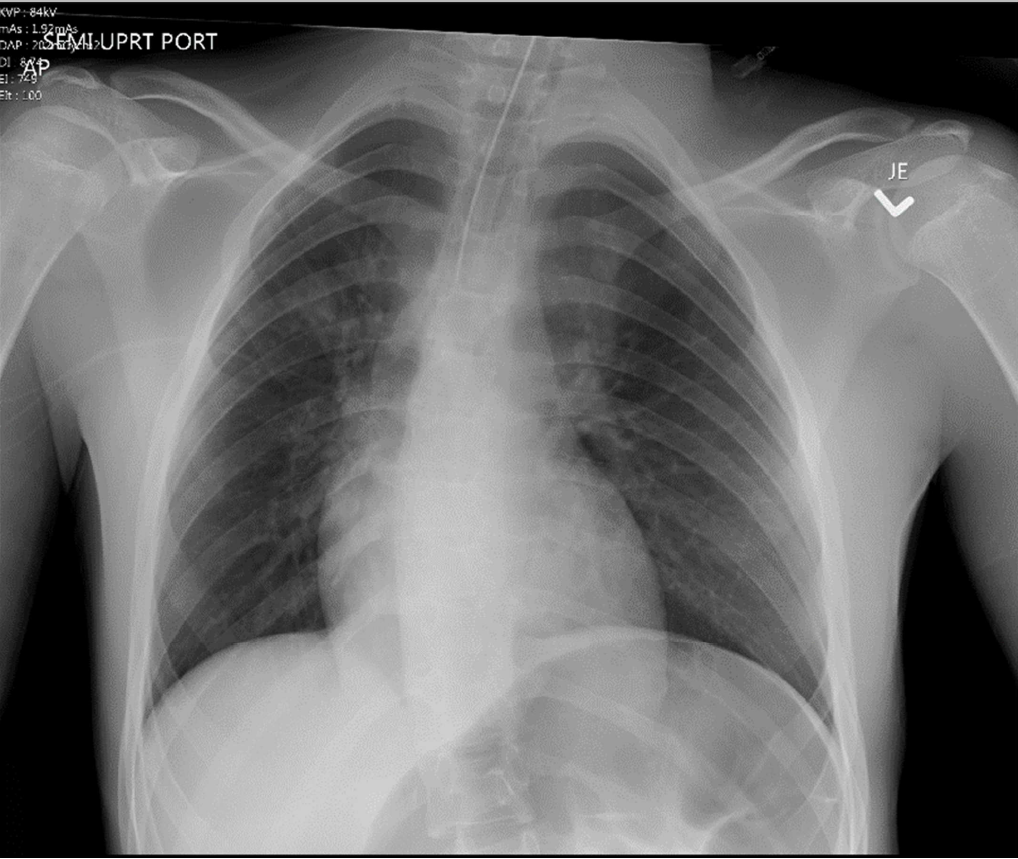


*Author owned: Dr. Corinne Shubin*

Supplement: Supplementary file 1 — Simulation Case.docxSimulation Equipment Preparation.docxSimulation Critical Action Checklist.docxSimulation ECG.docxSimulation Intubated CXR.docxSimulation Debriefing Guide.docxSimulation Teamwork and Communication Glossary.docxSimulation Didactic.pptxSimulation Evaluation Form.docx [file mep_2374-8265.10928-s001.zip › E. Simulation Intubated CXR.docx]
